# Characteristics of adolescents aged 15-19 years living with vertically and horizontally acquired HIV in Nampula, Mozambique

**DOI:** 10.1371/journal.pone.0250218

**Published:** 2021-04-26

**Authors:** Chloe A. Teasdale, Kirsty Brittain, Allison Zerbe, Claude Ann Mellins, Joana Falcao, Aleny Couto, Eduarda Pimentel De Gusmao, Mirriah Vitale, Bill Kapogiannis, Teresa Beatriz Simione, Landon Myer, Joanne Mantell, Christopher Desmond, Elaine J. Abrams

**Affiliations:** 1 Department of Epidemiology and Biostatistics, CUNY Graduate School of Public Health & Health Policy, New York, New York, United States of America; 2 ICAP at Columbia University, Mailman School of Public Health, New York, New York, United States of America; 3 Department of Epidemiology, Mailman School of Public Health, Columbia University, New York, New York, United States of America; 4 Division of Epidemiology & Biostatistics, School of Public Health & Family Medicine, University of Cape Town, Cape Town, South Africa; 5 Centre for Infectious Disease Epidemiology & Research, School of Public Health & Family Medicine, University of Cape Town, Cape Town, South Africa; 6 Department of Psychiatry, HIV Center for Clinical and Behavioral Studies, New York State Psychiatric Institute and Columbia University Irving Medical Center, New York, New York, United States of America; 7 National STI, HIV/AIDS Control Program, Maputo, Mozambique; 8 Eunice Kennedy Shriver National Institute of Child Health and Human Development, Bethesda, Maryland, United States of America; 9 Centre for Rural Health, University of KwaZulu Natal, Durban, South Africa; 10 Department of Pediatrics, Vagelos College of Physicians and Surgeons, Columbia University, New York, New York, United States of America; 1. IRCCS Neuromed 2. Doctors with Africa CUAMM, ITALY

## Abstract

**Background:**

Adolescents living with HIV (ALHIV) 15–19 years of age are a growing proportion of all people living with HIV globally and the population includes adolescents with vertically acquired HIV (AVH) and behaviorally acquired HIV (ABH).

**Methods:**

We conducted a survey to measure sociodemographic characteristics, educational status, health history, and antiretroviral therapy (ART) adherence among a convenience sample of ALHIV at three government health facilities in 2019 in Nampula, Mozambique. ALHIV 15–19 years on ART, including females attending antenatal care, were eligible. Routine HIV care data were extracted from medical charts. Classification of ALHIV by mode of transmission was based on medical charts and survey data. ALHIV who initiated ART <15 years or reported no sex were considered AVH; all others ABH. Frequencies were compared by sex, and within sex, by mode of transmission (AVH vs. ABH) using Chi-square, Fishers exact tests and Wilcoxon rank-sum tests.

**Results:**

Among 208 ALHIV, 143 (69%) were female and median age was 18 years [interquartile range (IQR) 16–19]. Just over half of ALHIV (53%) were in or had completed secondary or higher levels of education; the most common reason for not being in school reported by 36% of females was pregnancy or having a child. Of all ALHIV, 122 (59%) had VL data, 62% of whom were <1000 copies/mL. Almost half (46%) of ALHIV reported missing ARVs ≥ 1 day in the past month (62% of males vs. 39% of females; p = 0.003). Just over half (58%) of ALHIV in relationships had disclosed their HIV status: 13% of males vs. 69% of females (p<0.001). Among sexually active males, 61% reported using a condom at last sex compared to 26% of females (p<0.001). Among female ALHIV, 50 (35%) were AVH and 93 (65%) were ABH, 67% of whom were not in school compared to 16% of ABH, (p<0.001).

**Discussion:**

Data from our study underscore the high level of deprivation among ALHIV enrolled in HIV care in Mozambique, as well as important disparities by sex and mode of transmission. These data can inform the development of effective interventions for this complex and important population.

## Introduction

Adolescents living with HIV (ALHIV), ages 15–19 years, are a growing proportion of all people living with HIV globally and the vast majority (88%), approximately 1.5 million, live in sub-Saharan Africa (SSA) [1,2]. Compared to adults and children, ALHIV have worse outcomes across the HIV care continuum, with lower testing, antiretroviral therapy (ART) coverage, and viral suppression rates [3–6]. Data from the Population-based HIV Incidence Assessments (PHIA) conducted in Eswatini, Lesotho, Malawi, Zambia and Zimbabwe found that only 56% (95% CI 50–62) of ALHIV 15–19 years were aware of their HIV-positive status; among ALHIV with known status, 50% (95% CI 44–56) were on ART and only 35% (95% CI 30–41%) on ART had a suppressed HIV viral load (VL<50 copies/mL) [7]. As a result of the low coverage and uptake of services, HIV is the leading cause of death for adolescents 10–19 years of age in SSA [1]. Mozambique is one of the ten countries with the highest burden of adolescent HIV globally, with approximately 140,000 ALHIV and 7% HIV prevalence among females 15–19 years of age, yet there have been few studies describing the characteristics of this population [8,9].

The ALHIV population is comprised of adolescents with vertically acquired HIV-transmission (AVH) and behaviorally acquired HIV (ABH) [1,8,10]. While the population of AVH has increased as a result of efforts over recent decades to improve pediatric diagnosis and expand access to ART leading to better survival among children living with HIV [11,12], the increasing population of ABH is driven by alarmingly high HIV infection rates among young women 15–24 years of age [13]. In SSA, adolescent and young women are up to six times more likely to acquire HIV compared to males the same age and they account for one of every five new HIV infections despite representing only 10% of the population [10,14].

Adolescence is a critical period of physical, emotional, and social development, characterized by heightened vulnerability and challenges which may impact adherence to HIV care and ART [15]. Mode of transmission may impact how adolescents navigate their HIV status and care. For many AVH, adolescence is when they learn their HIV status [16] which occurs in the context of emerging independence, including responsibility for their own care and disclosing their HIV status to peers and sexual partners [17–19]. In addition, AVH may need more complex clinical care as a result of late ART initiation as well as long-term use [20,21]. They may also experience medication fatigue and other adherence challenges which have been observed among adolescents with other chronic diseases [22–24]. ABH may face different barriers to achieving optimal treatment outcomes. As noted, ABH in SSA are predominantly young women who are more likely to experience gender-based violence and gender inequality [10]. In addition, many female ABH are identified as a result of pregnancies [25,26] which entail new responsibilities that can make engagement in care and adherence to ART more difficult [27,28].

Understanding and characterizing the ALHIV population in SSA with consideration of sex and mode of transmission may aid in the development and implementation of more effective models of HIV care and targeted interventions to improve outcomes; however, few studies of ALHIV from SSA have disaggregated results in this way [29–31]. We report data from a survey of ALHIV 15–19 years of age enrolled in HIV care in Nampula, Mozambique, in order to provide a more robust picture of the demographic, biomedical, psychosocial and behavioral characteristics of this key population based on sex and likely mode of transmission.

## Methods

The survey data reported in this analysis were collected during the first phase of the CombinADO study (UG3HD096926) which will test a multicomponent intervention to improve HIV care and treatment outcomes for ALHIV in Mozambique [32].

### Participants and procedures

The study was conducted at three government health facilities from June through December 2019 in the city of Nampula in northern Mozambique. All ALHIV 15–19 years of age who were enrolled in HIV care at the health facilities, including females attending antenatal care, were considered eligible to participate. Study staff reviewed facility reports to estimate the number of eligible participants; facility nurses provided information to ALHIV at routine care visits and referred them to study staff for more information and potential enrollment. Informed consent was obtained from ALHIV who were aged 18–19 years or emancipated, while assent was obtained from ALHIV aged 15–17 years with consent from their adult caregivers. Participants were given transportation reimbursement equivalent to $5 USD following survey completion. Study staff administered the surveys in Portuguese via electronic tablets to ALHIV in private spaces in health facilities.

### Measures

The quantitative survey was guided by Bronfenbrenner’s social-ecological model (SEM) addressing individual-level characteristics and health behaviors, interpersonal factors as well as higher level structural and contextual risk factors that may influence retention in care, ART adherence, and HIV viral suppression (S1 Fig) [33]. Individual-level factors measured in the survey included demographic characteristics of the adolescent, their caregivers and households, educational and employment status, as well as medical history, substance use, health beliefs and knowledge, ART adherence, and readiness for independent health care. The interpersonal domain covered relationship/marital status, sexual behavior, HIV disclosure, community engagement and pregnancy. Data collected on the institutional/health system characteristics are not reported in this analysis.

The survey included validated scales that were adapted for adolescents and translated into Portuguese and back-translated into English using recommended procedures [34]. Self-reported adherence was assessed using a tool developed by Wilson and colleagues which has been validated in the United States and South Africa [35]. The tool measures adherence reported over the past 30 days with three questions on: number of days with a missed ART dose, frequency with which respondents took ART the way they were supposed to and rating of how good a job they did taking their medications the way they were supposed to, the latter two items rated on a 5-point Likert scale). Self-reported adherence over the past 30 days was calculated for each participant by re-coding and weighting each item and then calculating the score ranging from 0–100% [35,36]. We also measured knowledge about HIV using ‘true’/’false’/‘don’t know’ responses to statements about HIV transmission, viral load measurements and ART adherence using questions adapted from previous HIV surveys [2,37]. For the analysis, we dichotomized responses into incorrect/‘don’t know’ and correct for each question. To measure stigma and self-perceived stigmatization, we adapted the Social Impact Scale which measures responses across four dimensions of internalized and externalized stigma: social rejection, financial insecurity, internalized shame, and social isolation [38]. Participants were asked to indicate their agreement with statements about how they have been treated and how they feel about their HIV-positive status (for the presentation of results, we combined ‘strongly agree’ with ‘agree’ and ‘strongly disagree’ with ‘disagree’). As this study was conducted with adolescents who are less likely than adults to have disclosed their HIV-positive status to others [39,40] and thus may not have experienced stigma due to non-disclosure, an additional response option indicating non-disclosure (yes/no) was added to each item on the scale to help us differentiate lack of stigma experiences due to lack of disclosure from lack of stigma experiences. The London Measure of Unplanned Pregnancy (LMUP), which has been validated in sub-Saharan Africa, was used to collect information from women who were currently pregnant [41]. Mode of transmission was not collected directly from ALHIV in this study and is not recorded routinely in medical charts; thus, we estimated mode of transmission based on available information. ALHIV were considered AVH if they initiated ART before the age of 15 years (based on self-report or medical chart), or if they reported never having vaginal intercourse; all other ALHIV were considered ABH (S2 Fig).

Study staff administered the surveys in Portuguese via electronic tablets to ALHIV in private spaces in health facilities. Routine data on participant HIV care and treatment history were extracted from medical charts, including HIV diagnosis and ART initiation dates, ART regimens and viral load (VL) measures. The protocol was approved by the Columbia University Irving Medical Center (CUIMC) Institutional Review Board (IRB) and the Comité Nactional de Bioética para Saúde of the Ministry of Health in Mozambique.

### Data analysis

We present descriptive data on the characteristics of ALHIV 15–19 years of age. We also examined characteristics according to sex and estimated mode of transmission (vertical vs. behavioral). Frequencies were compared by sex groups (all males compared to all females) and, among male and females separately, by mode of transmission (AVH vs. ABH) using Chi-square and Fishers exact tests for categorical and Wilcoxon rank-sum tests for continuous variables; p-values are presented for the comparison across all levels of multi-level categorical variables (for questions with multiple response options, binary variables were created for each option).

## Results

An estimated 310 ALHIV 15–19 years of age and active on ART were registered at the two facilities where the study was initially implemented. A total of 296 ALHIV (81 male, 215 female) were referred by site nurses for screening. Of these, 233 (60 male, 173 female) were eligible and 195 enrolled, including 49 males (82%) and 146 females (84%). Due to the low number of males enrolled, a third health facility was added that had 31 male ALHIV on ART, 18 of whom were referred for screening and all 18 were eligible and enrolled for a total of 213 participants. Data from 5/213 participants were excluded from this analysis because the participants were not aware of their HIV-positive status.

### Individual-level characteristics (Tables 1 & 2, Fig 1)

**Fig 1:**
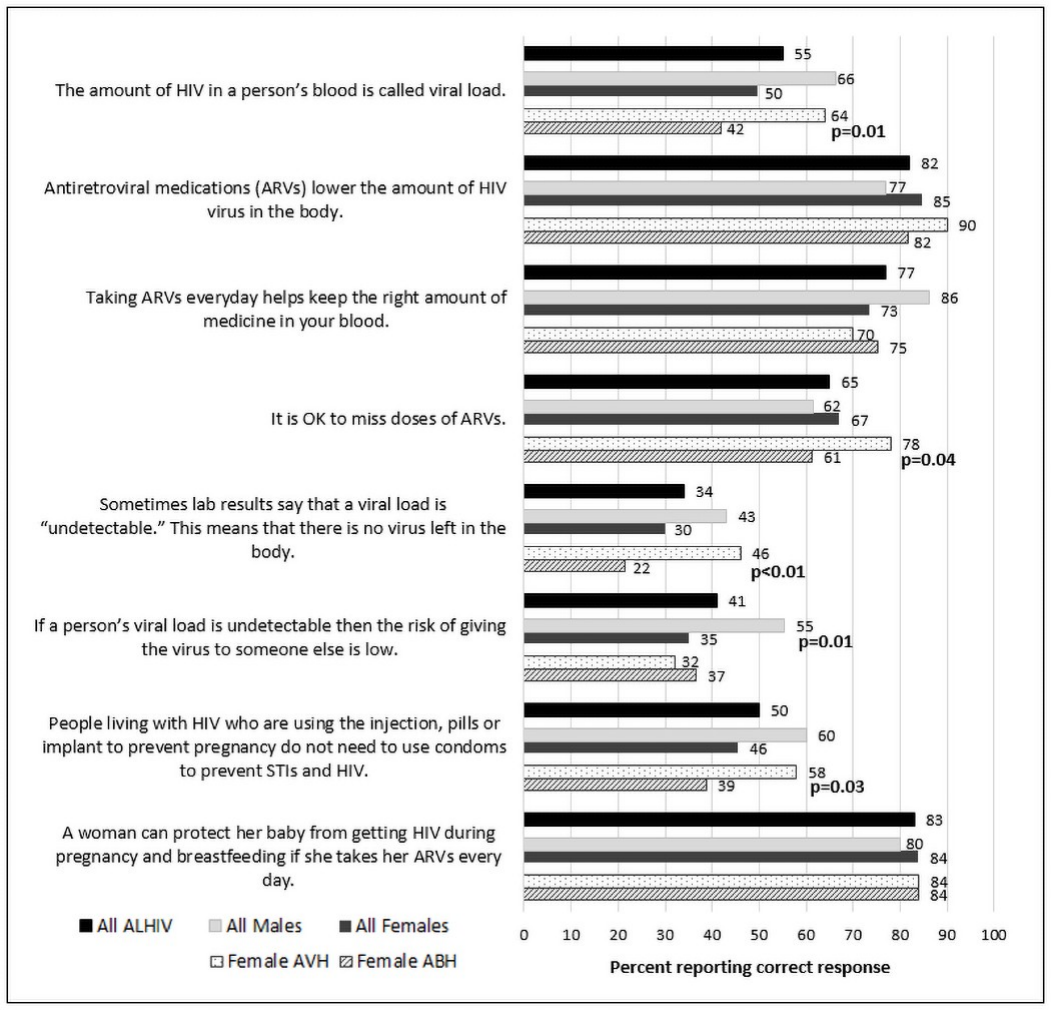
Proportion of ALHIV 15–19 years answering HIV knowledge questions correctly by sex and mode of transmission (vertically vs. behaviorally acquired HIV), Nampula, Mozambique, 2019 (N = 208).

**Table 1:** Demographic characteristics of adolescents living with HIV 15–19 years of age enrolled in care at three health facilities in Nampula, Mozambique, 2019 (N = 208).

	Total		Males		Females		
	N	*%*	N	*%*	N	*%*	p-value
	208	*100*	65	*31*	143	*69*	
**Age, median *(IQR)***	18 *(16–19)*		17 *(15–18)*		18 *(17–19)*		<0.001
**Age at ART initiation, median *(IQR)***	16 *(12–18)*		13 *(9–16)*		17 *(15–18)*		<0.001
**Don’t know**	23	*11*	5.0	*8*	18	*13*	0.35
**Household characteristics & resources**							
**Muslim**	77	*37*	28	*43*	49	*34*	0.50
**Catholic**	71	*34*	20	*31*	51	*36*	
**Protestant/Evangelic**	52	*25*	16	*25*	36	*25*	
**Other/no religion**	8	*4*	1	*2*	7	*5*	
**Primary caregiver**							
**Mother**	67	*32*	28	*43*	39	*27*	<0.001
**Father**	26	*13*	10	*15*	16	*11*	
**Sister/brother**	19	*9*	9	*14*	10	*7*	
**Aunt/uncle**	19	*9*	8	*12*	11	*8*	
**Grandmother/grandfather**	13	*6*	7	*11*	6	*4*	
**Partner (husband, boyfriend)**	31	*15*	0	*0*	31	*22*	
**Self**	30	*14*	1	*2*	29	*20*	
**Other**	3	*1*	2	*3*	1	*1*	
**Primary provider of financial support**							
**Mother**	46	*22*	14	*22*	32	*22*	<0.001
**Father**	46	*22*	21	*32*	25	*18*	
**Other family**	43	*21*	24	*37*	19	*13*	
**Partner**	59	*28*	0	*0*	59	*41*	
**Self**	5	*2*	1	*2*	4	*3*	
**Other**	9	*4*	5	*8*	4	*3*	
**Household facilities**							
**Inside toilet**	69	*33*	45	*70*	24	*17*	<0.001
**Running water**	48	*23*	30	*47*	18	*14*	<0.001
**Electricity**	158	*76*	55	*86*	103	*72*	0.03
**Adolescent or caregiver can afford**							
**Visit to the doctor when you are ill**	201	*97*	61	*95*	140	*98*	0.38
**Three meals a day**	164	*79*	47	*73*	117	*82*	0.17
**School fees (n = 128)**	123	*96*	54	*98*	69	*95*	0.39
**All of the medicines needed**	191	*92*	56	*88*	135	*94*	0.09
**Not enough food ≥1 day past week**	49	*24*	31	*48*	18	*13*	<0.001
**Cell phone ownership**							
**Has own cell phone**	95	*46*	30	*46*	65	*46*	0.86
**Shares a cell phone**	56	*27*	16	*25*	40	*28*	
**No cell phone**	57	*27*	19	*29*	38	*27*	
**Has accessed the internet**	77	*37*	35	*54*	42	*29*	<0.01
**Frequency of internet**							
**Daily**	32	*42*	16	46	16	*38*	0.65
**Weekly**	33	*43*	15	43	18	*43*	
**Monthly or more**	12	*16*	4	11	8	*19*	
**Substance use (ever)**							
**Tobacco**	5	*2*	5	8	0	*0*	<0.01
**Alcohol**	26	*13*	14	22	12	*8*	0.01
**Marijuana**	3	*1*	3	5	0	*0*	0.03
**Education & employment**							
**Current school enrollment**							
**Primary (first and second)**	19	*9*	11	*17*	8	*6*	<0.001
**Secondary (first and second)**	106	*51*	43	*66*	63	*44*	
**Technical or vocational school**	2	*1*	1	*2*	1	*1*	
**University, college or other tertiary**	2	*1*	1	*2*	1	*1*	
**Not in school**	79	*38*	9	*14*	70	*49*	
**Highest grade completed (n = 79)**							
**None**	4	*5*	0	*0*	4	*6*	0.91
**Incomplete primary**	35	*44*	5	*56*	30	*43*	
**Primary education**	7	*9*	0	*0*	7	*10*	
**Incomplete secondary**	27	*34*	3	*33*	24	*34*	
**Secondary education**	6	*8*	1	*11*	5	*7*	
**Reasons for not attending school**							
**Finished**	3	*4*	1	*11*	2	*3*	0.31
**Couldn’t pay school fee/uniforms**	20	*25*	5	*56*	15	*21*	0.04
**Stopped to help at home/get job**	9	*11*	0	*0*	9	*13*	0.59
**Too unwell**	6	*8*	1	*11*	5	*7*	0.53
**Parent/guardian died**	2	*3*	1	*11*	1	*1*	0.22
**Got married**	17	*22*	0	*0*	17	*24*	0.19
**Pregnancy/had child**	25	*32*	0	*0*	25	*36*	0.05
**Moved**	8	*10*	2	*22*	6	*9*	0.23
**Other**	14	*18*	1	*11*	13	*19*	1.00
**Employment status**							
**Currently employed**	7	*3*	1	*2*	6	*4*	0.66
**Previously employed**	16	*8*	4	*6*	12	*8*	
**Never employed**	185	*89*	60	*92*	125	*87*	
**Self-reported health status**							
**Current health status**							
**Excellent/very good**	47	*23*	14	*22*	33	*23*	0.01
**Good**	106	*51*	27	*42*	79	*55*	
**Fair**	35	*17*	20	*31*	15	*11*	
**Poor**	20	*10*	4	*6*	16	*11*	
**Past year**							
**Too sick to attend work/school ≥1 day**	75	*36*	35	*54*	40	*28*	<0.001
**≥1 night in hospital**	21	*10*	8	*12*	13	*9*	0.48
**TB diagnosis**	14	*7*	7	*11*	7	*5*	0.12
**Ever diagnosed or treated for STI**	33	*16*	3	*5*	30	*21*	<0.01
**Past 6 months symptoms reported sometimes or often**							
**Asthma, lung problems and trouble breathing for > 2 days**	44	*21*	15	*23*	29	*20*	0.65
**Bad cough**	108	*52*	36	*55*	72	*50*	0.50
**Night sweats**	72	*35*	19	*29*	53	*37*	0.26
**Ulcers in mouth or problems swallowing food**	47	*23*	13	*20*	34	*24*	0.55
**Diarrhea >2 days in a row**	94	*45*	32	*49*	62	*43*	0.43
**Weight loss/inability to put on weight**	93	*46*	25	*39*	68	*49*	0.16
**Smelly vaginal or penile discharge**	42	*20*	5	*8*	37	*26*	<0.01
**Medical chart data**							
**CD4 cell count measure past year**	27	*13*	11	*17*	16	*11*	0.25
**Median CD4 cell count *(IQR)***	644 *(508*, *803)*		579 *(363*, *694)*		734 *(567*, *841)*		0.14
**Viral load measure past year**	122	*59*	41	63	81	57	0.38
**Median log_10_ viral load *(IQR)***	1.7 *(1*.*7*, *4*.*1)*		2.9 *(1*.*7*, *4*.*3)*		1.7 *(1*.*7*, *3*.*6)*		0.21
**<50 copies/mL**	61	*50*	17	*42*	44	*54*	0.18
**<1000 copies/mL**	75	*62*	22	*54*	53	*65*	0.21
**Regimen (11 missing regimen data)**							
**TDF+3TC+EFV**	142	*72*	24	*39*	118	*87*	<0.001
**TDF+3TC+DTG**	30	*15*	20	*32*	10	*7*	
**2 NRTI+PI (ATZ/r, LPV/r)**	14	*7*	11	*18*	3	*2*	
**AZT+3TC+NVP**	8	*4*	5	*8*	3	*2*	
**Other**	3	*2*	2	*3*	1	*1*	

*TDF: Tenofovir, 3TC: Lamivudine, EFV: Efavirenz, DTG: Dolutegravir, NRT: Non-nucleoside reverse transcriptase inhibitors including AZT: Zidovudine or ABC: Abacavir, ATZ/r: Atazanavir/ritonavir, LPV/r: Lopinavir/ritonavir, NVP: Nevirapine.

**Table 2:** Self-reported health status, adherence and health autonomy among adolescents living with HIV 15–19 years of age care at three health facilities in Nampula, Mozambique, 2019 (N = 208).

	Total		Males		Females		
	N	%	N	%	N	%	p-value
	208	*100*	65	*31*	143	*69*	
**ART responsibility**							
**Frequency of ART pick-up**							
**Monthly**	161	*77*	42	*65*	119	*83*	0.01
**Every 3 months**	45	*22*	22	*34*	23	*16*	
**Other**	2	*1*	1	*2*	1	*1*	
**Takes one ARV pill per day**	155	*75*	38	*59*	117	*82*	<0.001
**Knows prescribed ARV names**	8	*4*	6	*9*	2	*1*	0.01
**Person responsible for administering ART**							1.00
**Self**	191	*92*	60	*92*	131	*92*	
**Caregiver**	10	*5*	3	*5*	7	*5*	
**Both self and caregiver**	7	*3*	2	*3*	5	*4*	
**Self-reported ART adherence**							
**Missed ARVs ≥ 1 day in past 30 days**	96	*46*	40	*62*	56	*39*	<0.01
**Took ART as instructed past 30 days**							
**Always**	113	*54*	46	*71*	67	*47*	<0.01
**Almost always**	69	*33*	11	*17*	58	*41*	
**Sometimes/usually**	19	*9*	8	*12*	11	*8*	
**Never/rarely**	7	*3*	0	*0*	7	*5*	
**Did a good job taking ART as instructed past 30 days**							
**Very poor/poor**	6	*3*	1	*2*	5	*4*	0.13
**Fair**	29	*14*	13	*20*	16	*11*	
**Good**	76	*37*	27	*42*	49	*34*	
**Very good/excellent**	97	*47*	24	*37*	73	*51*	
**Difficulty taking ARVs as instructed**							
**Not hard/not very hard**	184	*89*	62	*95*	122	*85*	0.10
**Somewhat hard**	18	*9*	2	*3*	16	*11*	
**Extremely hard/very hard**	6	*3*	1	*2*	5	*4*	
**3-item adherence score, median *(IQR)***	89 *(81–94)*		87 *(81*, *93)*		89 *(81*, *94)*		0.48
**Reported reasons for any missed ART doses past 30 days**							
**None missed**	111	*53*	25	*39*	86	*60*	<0.01
**Forgot**	63	*30*	25	*39*	38	*27*	0.08
**Different routine**	21	*10*	11	*17*	10	*7*	0.03
**No food to take with ARVs**	15	*7*	6	*9*	9	*6*	0.45
**Ran out**	8	*4*	5	*8*	3	*2*	0.11
**Unwell or vomiting**	6	*3*	4	*6*	2	*1*	0.08
**Didn’t like taste**	5	*2*	2	*3*	3	*2*	0.65
**Fed up or tired or taking ARVs**	4	*2*	2	*3*	2	*1*	0.59
**Did not want others to see ARVs**	3	*1*	1	*2*	2	*1*	1.00
**Readiness for independent care**							
**Keeps track of clinic appointments**							
**Always on own**	154	*74*	50	*77*	104	*73*	0.55
**With help from caregiver**	34	*16*	8	*12*	26	*18*	
**Caregiver always does**	20	*10*	7	*11*	13	*9*	
**Always attends clinic on own**	180	*87*	55	*85*	125	*87*	0.58
**Explain health issues to provider**							
**Always on own**	173	*83*	49	*75*	124	*87*	0.07
**With help from caregiver**	27	*13*	13	*20*	14	*10*	
**Caregiver always does**	7	*3*	2	*3*	5	*4*	
**N/A (doesn’t do)**	1	*1*	1	*2*	0	*0*	
**Tracks stock of own medications**							
**Always on own**	111	*53*	25	*39*	86	*60*	<0.001
**With help from caregiver**	34	*16*	14	*22*	20	*14*	
**Caregiver always does**	27	*13*	17	*26*	10	*7*	
**N/A (doesn’t do)**	36	*17*	9	*14*	27	*19*	
**Know when to take medication**							
**Always on own**	199	*96*	61	*94*	138	*97*	0.16
**With help from caregiver**	7	*3*	2	*3*	5	*4*	
**Caregiver always does**	2	*1*	2	*3*	0	*0*	

#### Individual and household characteristics

Among 208 ALHIV who were included in the analysis, 65 (31%) were males and 143 (69%) were female (Table 1). Median age of participants was 18 years [interquartile range (IQR) 16–19] and median age at ART initiation was 16 years [IQR 12–18]. Males were younger at ART initiation (13 years, IQR 9–16) compared to females (17 years, IQR 15–18) (p<0.001). A third of all ALHIV (32%) reported mothers as primary caregiver. Females were more likely to report partners (22%) or themselves (20%) as their primary caregivers whereas no males reported partners and 1 (2%) reported himself (p<0.001). While most ALHIV (76%) had electricity in the home, only 33% had inside toilets and 23% had running water. Significantly higher proportions of males had access to household facilities (Table 1). While almost all ALHIV reported having enough money to seek medical care (97%) and pay for medications (92%), 24% reported not having enough food in the home in the past week, with males more likely to report this than females (48% vs. 13%; p<0.001). Less than half (46%) of ALHIV had their own cell phone and only 37% had ever accessed the internet (54% of males vs. 29% of females, p = 0.001). Very few ALHIV reported ever using tobacco (2%), alcohol (13%) or marijuana (1%) (no females reported tobacco or marijuana use).

#### Education & employment

Just over half of ALHIV (53%) were in or had completed secondary or higher levels of education; 9% were in primary school and 38% were not in school (Table 1). For males, the primary reason for not completing school was not being able to pay fees (56%), while the most common reason reported by females was a pregnancy or having a child (36%). Only 3% of all ALHIV reported being currently employed; most (89%) reported never having been employed.

#### Self-reported health status

Most ALHIV reported excellent (23%) or good (51%) health status’ however, more males reported current health status as fair or poor compared to females (37% vs. 22%; p = 0.005) (Table 1). Sixteen percent of ALHIV reported ever having been diagnosed with a sexually transmitted infection (STI), 5% of males compared to 12% of females (p<0.001). Many ALHIV reported ailments in the past six months, including a bad cough (52%), weight loss (46%) or night sweats (45%).

#### Medical chart data

Only 122 (59%) ALHIV had VL data in medical charts (Table 1). Median log_10_ VL was 1.7 copies/mm^3^ (IQR 1.7–4.1) and 62% of ALHIV with a VL measure had <1000 copies/mL. Most ALHIV (72%) were on an ART regimen containing tenofovir (TDF), lamivudine (3TC), and efavirenz (EFV) and 15% received TDF+3TC+dolutegravir (DTG).

#### Health knowledge and beliefs

Responses to questions measuring knowledge about HIV transmission and treatment are shown in Fig 1. Most ALHIV understood how ARVs work (82%) and that they can prevent vertical transmission (83%); however, few provided correct responses to questions about viral load. More males than females knew what VL measures (66% males vs. 50% females; p = 0.03); that when VL is undetectable, transmission is less likely (55% males vs. 35% females; p = 0.006); and that they need to take ARVs every day (86% males vs. 73% females; p = 0.04).

#### Responsibility for ART administration

Most ALHIV (77%) picked up medication monthly rather than quarterly (Table 2). Almost all ALHIV (92%) reported being responsible for administering their own ART. However, only 8 ALHIV (4%) knew the names of the ARVs they were prescribed.

#### Self-reported ART adherence

Almost half (46%) of ALHIV reported missing ARVs at least one day in the past month with males more likely than females to report missed ARV doses (62% vs. 39%; p = 0.003) (Table 2). Overall, 47% of ALHIV reported ‘very good’ or ‘excellent’ taking of medication and 89% said it was ‘not hard’ or ‘not very hard’. The median 3-item adherence score for the past 30 days was 89% (IQR 81–94), with no difference by sex. The most commonly reported reasons for missing ARV doses in the past month was forgetting (30%), changes in routine (10%) and no food to take with medication (7%). A higher proportion of males reported adherence challenges resulting from ‘changes to routine’ compared to females (17% vs. 7%; p = 0.028).

### Interpersonal-level characteristics (Tables 3–5)

**Table 3:** Relationship status, HIV stigma/disclosure and community engagement among ALHIV 15–19 years of age enrolled in HIV care in Nampula, Mozambique, 2019 (N = 208).

	Total		Males		Females		
	N	*%*	N	%	N	%	p-value
	208	*100*	65	31	143	69	
**Current relationship**							
**Currently in a relationship**	120	*58*	23	35	97	68	<0.001
**Current relationship status**							<0.001
**Married and living together**	42	*35*	0	0	42	43	
**Married but not living together**	15	*13*	0	0	15	16	
**Not married but living together**	7	*6*	0	0	7	7	
**Not married and not living together**	53	*44*	23	100	30	31	
**Other**	3	*3*	0	0	3	3	
**Current relationship length, median *(IQR)* months**	12 *(6*,*24)*		5 *(2*,*12)*		12 *(7*,*24)*		0.01
**Age of partner, median *(IQR)* age**	22 *(19*,*25)*		16 *(15*,*18)*		23 *(21*,*26)*		<0.001
**Age at marriage (n = 57), median *(IQR)***	17 *(16*,*18)*		-		17 *(16*,*18)*		-
**Partner is parent of a participant’s child (n = 58)**	48	*83*	0	*0*	48	*83*	-
**Other sexual partners in last year (n = 111)**	17	*15*	6	*43*	11	*11*	<0.01
***HIV status disclosure***							
**Remembers age when first learned HIV status**	197	*95*	63	*97*	134	*94*	0.51
**Age learned HIV status, median *(IQR)***	16 (*14*, *18)*		14 *(13*, *15)*		17 *(15*, *18)*		<0.001
**How learned HIV status**							<0.01
**On own (no one told)**	5	*2*	1	*2*	4	*3*	
**Told by doctor/nurse**	86	*41*	18	*28*	68	*48*	
**Told by family at home**	23	*11*	14	*22*	9	*6*	
**Told by family at clinic**	92	*44*	31	*48*	61	*43*	
**Don’t remember/other**	2	*1*	1	*2*	1	*1*	
**Other household members with HIV**							
**Mother**	50	*24*	13	*20*	37	*26*	0.36
**Father**	20	*10*	4	*6*	16	*11*	0.32
**Brother/sister**	34	*16*	7	*11*	27	*19*	0.14
**Aunt/uncle**	20	*10*	11	*17*	9	*6*	0.02
**Grandmother/grandfather**	6	*3*	6	*9*	0	*0*	0.00
**Partner (wife/husband, boyfriend/girlfriend)**	15	*7*	1	*2*	14	*10*	0.04
**Other**	41	*20*	22	*34*	19	*13*	0.00
**Don’t know**	72	*35*	18	*28*	54	*38*	0.16
**Primary caregiver knows adolescent’s HIV status**							<0.001
**Yes**	140	*67*	62	*95*	78	*55*	
**No**	45	*22*	1	*2*	44	*31*	
**Don’t know**	23	*11*	2	*3*	21	*15*	
**Household members know adolescent’s HIV status**							0.02
**None**	21	*10*	1	*2*	20	*14*	
**Few**	121	*58*	40	*62*	81	*57*	
**Most all**	61	*29*	22	*34*	39	*27*	
**Don’t know**	5	*2*	2	*3*	3	*2*	
**Family outside household know adolescent’s HIV status**							0.87
**None**	85	*41*	25	*39*	60	*42*	
**Few**	93	*45*	29	*45*	64	*45*	
**Most all**	20	*10*	7	*11*	13	*9*	
**Don’t know**	10	*5*	4	*6*	6	*4*	
**Disclosed HIV status to current partner among those with partners (n = 120)**	70	*58*	3	*13*	67	*69*	<0.001
**Knows current partner’s HIV status (n = 120)**	56	*47*	3	*13*	53	*55*	<0.001
**Current partner’s HIV status (n = 56)**							0.56
**HIV-positive**	22	*39*	2	*67*	20	*38*	
**HIV-negative**	34	*61*	1	*33*	33	*62*	
**Friends know adolescent’s HIV status**							0.35
**None**	184	*89*	55	*85*	129	*90*	
**Few**	16	*8*	8	*12*	8	*6*	
**Most all**	2	*1*	0	*0*	2	*1*	
**Don’t know**	6	*3*	2	*3*	4	*3*	
**Teachers know adolescent’s HIV status among those in school (n = 127)**							0.40
**None**	121	*95*	52	*93*	69	*97*	
**Some**	6	*5*	4	*7*	2	*3*	
**Community engagement**							
**Member of a youth organization(s)**							
**Gospel choir/church group**	37	*18*	6	*9*	31	*22*	0.03
**Activist organization/youth association**	18	*9*	14	*22*	4	*3*	<0.001
**Sports team**	11	*5*	9	*14*	2	*1*	0.00
**Music/singing/arts performance group**	8	*4*	2	*3*	6	*4*	1.00
**Any above**	65	*32*	26	*41*	39	*28*	0.08
**Past year involved/participated in following**:							
**Cultural or religious organization**	71	*35*	24	*38*	47	*34*	0.61
**Sports or Recreation**	44	*22*	33	*52*	11	*8*	<0.001
**Performing Arts (theater, music, etc.)**	40	*20*	14	*22*	26	*19*	0.60
**Academic or Pre-Professional Society**	9	*4*	3	*5*	6	*4*	1.00
**Government or Political Organization**	4	*2*	0	*0*	4	*3*	0.31
**Community Based Organization**	5	*3*	2	*3*	3	*2*	0.65
**Media (newspaper, radio, TV, etc.)**	8	*4*	5	*8*	3	*2*	0.11
**Other**	1	*1*	0	*0*	1	*1*	1.00

**Table 4:** Sexual behaviors and condom use among ALHIV 15–19 years of age who have had vaginal intercourse in Nampula, Mozambique, 2019 (N = 140).

	Total		Males		Females		
	N	*%*	N	*%*	N	*%*	p-value
	140	*100*	33	*24*	107	*76*	
**Portion of all ALHIV in survey who had sex**	140	*67*	33	*51*	107	*75*	<0.01
**Sexual behavior history**							
**Age at first sex, median *(IQR)* age**	16	*15*, *17*	15	*14*, *16*	16	*15*, *17*	0.01
**Age of first sexual partner, median *(IQR)***	18	*15*, *20*	15	*14*, *16*	19	*18*, *21*	<0.001
**Forced first sex (all)**	30	*22*	8	*24*	22	*21*	0.69
**Forced first sex**							0.68
**Physically forced**	10	*33*	2	*25*	8	*36*	
**Pressured**	20	*67*	6	*75*	14	*64*	
**Reason for first sex**							
**Wanted to try it**	75	*54*	18	*55*	57	*53*	0.01
**Partner wanted to have sex**	23	*16*	8	*24*	15	*14*	
**To show love/to feel loved**	23	*16*	2	*6*	21	*20*	
**Pressure from friends**	6	*4*	3	*9*	3	*3*	
**For money or gifts**	2	*1*	2	*6*	0	*0*	
**Wanted to have a baby**	2	*1*	0	*0*	2	*2*	
**Other/don’t know**	9	*6*	0	*0*	9	*8*	
**Total number of sex partners, median *(IQR)***	2	*1*, *3*	2	*1*, *4*	2	*1*, *3*	0.34
**Number sex partners last 12 months, median *(IQR)***	1 *(1*,*1)*		1 *(0*, *2)*		1 *(1*,*1)*		*0*.*16*
**Sex in exchange for money or gifts**	20	*14*	4	*12*	16	*15*	0.78
**Sources of information about sex and reproductive health**							
**Friends**	67	*48*	19	*58*	48	*45*	0.20
**Someone at the clinic**	67	*48*	14	*42*	53	*50*	0.48
**School**	37	*26*	15	*46*	22	*21*	0.01
**Internet**	24	*17*	14	*42*	10	*9*	<0.001
**Caregiver**	12	*9*	3	*9*	9	*8*	1.00
**The media**	11	*8*	2	*6*	9	*8*	1.00
**Older siblings**	11	*8*	8	*24*	3	*3*	<0.001
**Other**	9	*6*	1	*3*	8	*8*	0.69
**Condom use**							
**Condom used last sex**	47	*34*	20	*61*	27	*26*	<0.001
**Reasons for not using condom last sex act (n = 93)**							
**Partner refused**	60	*65*	1	*8*	59	*74*	<0.001
**Did not have a condom**	20	*22*	7	*54*	13	*16*	<0.01
**Felt safe without a condom**	7	*8*	0	*0*	7	*9*	0.59
**Wanted to become pregnant or get my partner pregnant**	6	*7*	0	*0*	6	*8*	0.59
**Other**	11	*12*	5	*39*	6	*8*	0.01
**Condom use**							
**Always**	26	*19*	15	*46*	11	*10*	<0.001
**Sometimes**	47	*34*	8	*24*	39	*37*	
**Never**	64	*46*	9	*27*	55	*51*	
**Don’t remember**	3	*2*	1	*3*	2	*2*	
**Reasons for condom use over past year (n = 140)**							
**Prevent pregnancy**	35	*25*	11	*33*	24	*22*	0.21
**Prevent HIV transmission**	29	*21*	10	*30*	19	*18*	0.12
**Prevent getting infected with STIs**	20	*14*	8	*24*	12	*11*	0.06
**Prevent reinfection with HIV**	13	*9*	4	*12*	9	*8*	0.51
**Other/don’t know**	33	*24*	4	*12*	29	*27*	0.10
**No condom use last year**	52	*37*	11	*33*	41	*38*	0.60

**Table 5:** Pregnancy status and history among female ALHIV 15–19 years of age in Mozambique who had ever been pregnant (N = 84).

	All		AVH		ABH		p-value
	N	*%*	N	*%*	N	*%*	
	84	*100*	7	*8*	77	*92*	
**Proportion of all females with history of pregnancy**		*59*		*14*		*54*	
**Total number of pregnancies (including current)**							1.00
**1**	61	*73*	5	*71*	56	*73*	
**2**	20	*24*	2	*29*	18	*23*	
**3**	3	*4*	0	*0*	3	*4*	
**Number of children given birth to who are living (n = 58)**							1.00
**0**	6	*10*	1	*25*	5	*9*	
**1**	45	*78*	2	*50*	43	*80*	
**2**	7	*12*	1	*25*	6	*11*	
**Number of biological children living with adolescent (n = 58)**							0.21
**0**	7	*12*	1	*25*	6	*11*	
**1**	45	*78*	2	*50*	43	*80*	
**2**	6	*10*	1	*25*	5	*9*	
**Number biological children tested HIV-positive (n = 58)**							1.00
**0**	46	*79*	4	*100*	42	*78*	
**1**	7	*12*	0	*0*	7	*13*	
**2**	1	*2*	0	*0*	1	*2*	
**Unknown**	4	*7*	0	*0*	4	*7*	
**Age at most recent pregnancy, median *(IQR)***	17 *(16*,*18)*		17 *(15*,*17)*		17 *(16*,*18)*		0.05
**Adolescent trying to get pregnant at time of last pregnancy**	58	*70*	4	*57*	54	*71*	0.53
**Currently breastfeeding**	37	*44*	1	*14*	36	*47*	0.13
							
**Currently pregnant**	23	*27*	2	*29*	21	*27*	1.00
**In the month that became pregnant…**.							0.17
**Self/partner not using contraception**	21	*91*	1	*50*	20	*95*	
**Self/partner using contraception sometimes**	1	*4*	1	*50*	0	*0*	
**Self/partner used but knew method had failed at least once**	1	*4*	0	*0*	1	*5*	
**Self/partner always used contraception**	0	*0*	0	*0*	0	*0*	
**Timing of pregnancy**							0.68
**Right time**	10	*44*	1	*50*	9	*43*	
**Ok, but not quite right time**	8	*35*	0	*0*	8	*38*	
**Wrong time**	5	*22*	1	*50*	4	*19*	
**Pregnancy intentions at time of pregnancy**							1.00
**Intended to get pregnant**	11	*48*	1	*50*	10	*48*	
**Changing intentions**	3	*13*	0	*0*	3	*14*	
**Did not intend to get pregnant**	9	*39*	1	*50*	8	*38*	
**Intentions to have baby at time of pregnancy**							0.46
**Wanted to have a baby**	12	*52*	1	*50*	11	*52*	
**Mixed feelings about having a baby**	6	*26*	0	*0*	6	*29*	
**Did not want to have a baby**	5	*22*	1	*50*	4	*19*	
**Currently receiving antenatal care**	22	*96*	1	*50*	21	*100*	0.09
**Health facility—pre-natal clinic**	8	*36*	1	*100*	7	*33*	0.36
Health facility—Adolescent services (SAAJ*)	14	*64*	0	*0*	14	*67*	
**Plans to delivery baby in**							1.00
**Health facility/hospital**	22	*96*	2	*100*	20	*95*	
**I don’t know**	1	*4*	0	*0*	1	*5*	
**Plans to continue taking ART after delivery**	23	*100*	2	*100*	21	*100*	-
**Plans to continue taking ART after stops breastfeeding**	22	*96*	2	*100*	20	*95*	1.00

*SAAJ Servicios Amigos dos Adolescentes.

#### Current relationship

Among 208 ALHIV, 121 (58%) reported a current relationship (35% of males and 69% of females; p<0.001) (Table 3). No males in relationships were married or cohabitating whereas more than half of females in relationships were married (59%) (p<0.001). Most males reported current relationships with other adolescents (median partner age: 16 years, IQR 15–18), whereas females were more likely to have adult partners (median partner age: 23 years, IQR 21–26) (p<0.001).

#### HIV status disclosure

Among all ALHIV, median age when they learned their HIV status was 16 years [IQR 14–18]; males were younger at HIV disclosure (14 years, IQR 13–15) compared to females (17 years, IQR 15–18) (p<0.001) (Table 2). Two-thirds (67%) of ALHIV reported that their primary caregiver knew the adolescent’s status (95% of males vs. 55% of females, p<0.000). Just over half of the 121 ALHIV (58%) with partners had disclosed to their partner; 13% of males vs. 69% of females (p<0.001). Less than half (47%) of ALHIV in relationships reported knowing their partner’s HIV status (13% of males and 55% of females, p<0.001). Most ALHIV (89%) reported that none of their friends knew they were living with HIV.

#### Community engagement

A third (32%) of all ALHIV reported involvement in youth organizations, including choirs or church groups, activist or youth associations, sports teams, or other arts groups (Table 3). Thirty-five percent reported cultural or religious organization participation and 22% reported participation in sports.

#### Social impact

**Fig 2:**
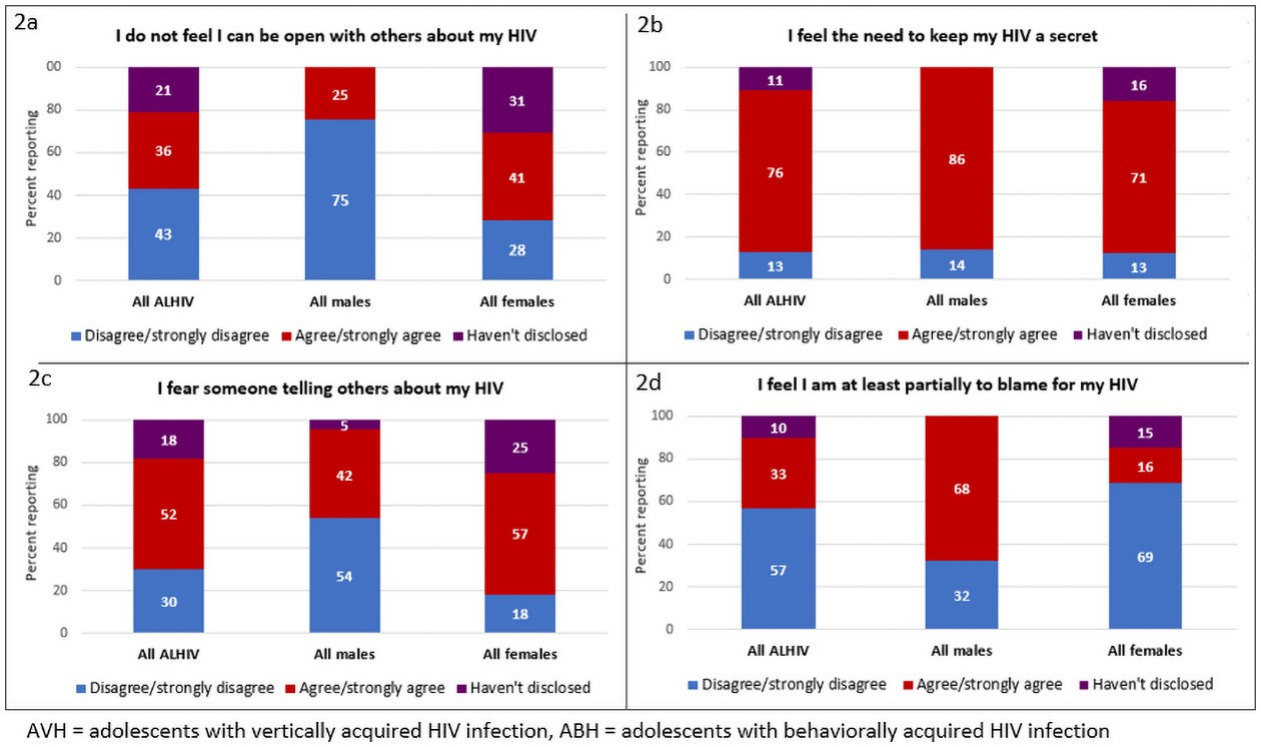
a-d. Responses to items on the Social Impact Scale from Mozambican ALHIV 15–19 years by sex and mode of HIV infection (N = 208).

On 8 out of the 15 Social Impact Scale items, more than 50% of females reported that they had not disclosed their HIV-positive status (S4 Table). Fig 2a–2d show responses to the items where the highest proportion of participants had disclosed. Among all ALHIV, 76% reported that they needed to keep their HIV a secret (86% of males and 71% of females, p<0.001) and 52% feared someone telling others about their HIV status (42% of males and 57% of females, p<0.0001) (Fig 2). A higher proportion of males (68%) than females (16%) agreed with the statement that they were partially to blame for their HIV (p<0.0001).

#### Sexual behavior

Among the 140 (66%) ALHIV who reported having had sexual intercourse, age at first sex was 16 years [IQR 15–17]; 15 years [IQR 14–16] for males and 16 years [IQR 15–17] for females (p = 0.006) (Table 4). Whereas males reported first sex with partners of the same age (median 15 years, IQR 14–16), first partners for females were older (19 years, IQR 18–21) (p<0.001). Overall, 22% of ALHIV reported their first sexual experience was forced, and among those, 33% said it was physically forced. Among all ALHIV, 14% reported ever having sex in exchange for money or gifts. More than half of sexually active males (61%) reported using a condom at their last sexual encounter, whereas only 26% of females reported condom at last sex (p<0.001) (Table 4).

#### Pregnancies and children

Among 143 female ALHIV, 84 (59%) had ever been pregnant (Table 5). Of 58 who had given birth to a child that was living, eight children (14%) were reported to have tested HIV-positive. The median age at the most recent pregnancy was 17 years (IQR 16–18) overall. At the time of the survey, 23 (27%) females were currently pregnant and 37 (44%) were breastfeeding. Among those who were pregnant, 91% said that in the month she became pregnant neither she nor her partner were using contraception. When asked about intentions at the time of the pregnancy, 39% said it was unintended.

### Analysis by estimated mode of transmission

We also examined characteristics according to estimated mode of transmission within sex. Among female ALHIV, 50 (35%) were AVH and 93 (65%) were ABH (S1 Table). Female ABH were older at treatment initiation, 18 years (IQR: 17–19) and at study, also 18 years (IQR: 18–19) compared to AVH who were 12 years (IQR: 9–16) at ART initiation and 16 years (IQR: 15–18) at study (p<0.001; p<0.001). A higher proportion of female ABH reported partners (30%) or themselves (20%) to be primary caregivers compared to female AVH (6% and 2%, respectively) (p<0.001). Significantly lower proportions of female ABH compared to AVH had an inside toilet (11% vs. 28%, p = 0.008) or running water (5% vs. 26%, p = 0.001). Only 19% of female ABH had ever accessed the internet (compared to 48% of AVH; p<0.001) and 67% were not in school (compared to 16% of AVH, p<0.001). Among female ABH not in school, 7% reported no schooling and 42% reported incomplete primary school (S1 Table). Female ABH also had less knowledge about HIV, particularly with regard to VL, and only 61% of ABH knew that they should not miss any doses of ARVs (compared to 78% of AVH, p = 0.04) (Fig 1). Based on self-reported adherence, a higher proportion of female ABH (19%) compared to AVH (2%) reported sometimes or never/rarely taking their ARVs over the past month (p = 0.04) (S2 Table). AVH females were more likely than ABH to have had a VL measurement, but rates of suppression were similarly low in both groups (51% vs. 57%, < 50 copies/mL). Female ABH were more likely to be in a relationship compared to female AVH (85% vs. 36%; p<0.0001) and 66% of ABH were married, whereas only 28% of AVH were (p = 0.03) (S3 Table). A higher proportion of female ABH (75%) had disclosed to their partners and were much less likely to report having used a condom at the last sex act (20%) compared to AVH, 44% of whom had disclosed to partners (p = 0.01) and 62% had used a condom at last sex (p = 0.004) (S5 Table).

Among male ALHIV, the majority (83%) were AVH and only 11 (17%) were ABH (S1 Table). Given the small sample size of ABH, there were few statistically significant differences observed according to mode of transmission for males. Similar to females, the male AVH were younger at treatment initiation, 12 years (IQR: 8–14), and study, 16 years (IQR: 15–18), compared to male ABH who were 17 years (IQR: 16–18) at ART initiation and 18 years (IQR: 17–19) at study (p<0.001; p<0.0001) (Table 1). A higher proportion of male ABH knew the names of their prescribed ARVs compared to AVH (36% vs. 4%, p = 0.006) (S2 Table).

## Discussion

Our survey describes the socio-demographic, behavioral and psychosocial characteristics of ALHIV 15–19 years of age engaged in HIV services in Mozambique. It highlights the significant challenges faced by this highly vulnerable population. Overall, study participants were poor, had limited access to household facilities, and low cellphone ownership and internet access, as well as low levels of educational attainment and employment. Self-reported adherence was high (89% overall); however, almost half of ALHIV reported at least one missed ARV dose in the past 30 days and, among those with viral load test results, viral suppression rates were low. ALHIV in the study also demonstrated limited knowledge and understanding of HIV and ART, with only 4% able to name the ARVs they were prescribed. Very few ALHIV had disclosed their HIV status to friends or to sexual partners. Most ALHIV were sexually active, but few reported consistent condom use and 20% reported forced sex.

Taken together, these characteristics highlight the high degree and breadth of unmet needs in multiple areas among ALHIV. Our study was conducted in Nampula, Mozambique, which is an economically disadvantaged city in one of the poorest countries in the world [42]. However, reports from other countries in SSA have also shown poverty to be significant among ALHIV, including a study in South Africa of over 1,000 ALHIV 10–19 years of age in which 68% lacked at least one basic necessity [30], as well as a qualitative study in Kenya in which ALHIV identified poverty as a major challenge to remaining in care [43]. Despite poverty being complex and beyond the scope of traditional medical care, targeted efforts to improve the economic status of ALHIV may be warranted. Although data are limited, economic empowerment interventions have shown some success in improving treatment outcomes in ALHIV and should be considered for inclusion in multicomponent interventions [44–46]. Other interventions at both the patient and health system levels have shown mixed results with regard to improving care outcomes in ALHIV [47,48], however there have been some promising findings from targeted adolescent-friendly services including expanded clinic hours and training for providers on adolescent care [44].

Stigma and fear about disclosure of HIV status were also major concerns for the ALHIV in our study as has been observed in other settings [39,49]. Stigma is a critically important factor to address with ALHIV as it has been associated with lack of retention in HIV services and poor adherence [50,51]. Assisting ALHIV with disclosure of HIV status to peers may be beneficial for reducing self-perceived stigma and building social support networks which can improve retention and ART adherence [39,50]. The low proportion of ALHIV in our study reporting disclosure to sexual partners, as well as condom use at last sex, along with the high rates of sexual activity and forced sex, have also been observed in other studies of ALHIV in SSA [18,52,53]. These data underscore the urgent need to strengthen sexual and reproductive health care for this population, and to provide support services for those who have been victims of gender-based sexual violence.

Our analysis also sheds new light on similarities and differences between males and females and likely mode of HIV transmission which can inform health and psychosocial intervention strategies aimed at improving the health of ALHIV. The majority (68%) in the cohort of ALHIV were female, and two-thirds (65%) were behaviorally infected. There were fewer males overall and unlike female ALHIV, most males (83%) had vertically acquired HIV. Our data are consistent with previous findings showing higher proportions of females among the ALHIV population in SSA which reflects disproportionately higher HIV acquisition as well as higher engagement in care among young women compared to men of similar age [29,54,55]. Despite previous documentation of sex differences among ALHIV [3,29,31,56], only a few studies have described this population according to mode of transmission in SSA where the majority of this population reside [30,31]. As such, our findings present important and novel data characterizing ALHIV, which will contribute to the design of more effective services globally for this group.

While our data illustrate the high level of deprivation and challenges faced by the ALHIV population overall, we also observed differences by sex and mode of HIV transmission. It should be noted that male study participants were almost entirely AVH, whereas females were primarily ABH, making it difficult to distinguish differences due to sex vs. mode of transmission. As expected, ABH of both sexes were older, had been on ART for less time and were less likely to be in school compared to AVH. Overall males were more likely than females to report alcohol use, and among ALHIV in relationships, ABH reported older partners and were more likely to have disclosed to their partners. Males overall were more likely than females to report missed ARV doses; however, using the three-item self-reported adherence scale, there were no differences across groups and all ALHIV in this study reported high adherence. The high levels of self-reported adherence may be due in part to social desirability which has been found in other studies [57–59]. Sex differences in self-reported adherence have also been previously reported among adults with men being more likely to report missed doses [60]. However, while reported adherence was relatively good, viral suppression rates were poor and did not differ by mode of transmission or sex. With regard to stigma, females were more likely to report needing to conceal their HIV status and reported more concern about inadvertent disclosure, while high proportions of males, both AVH and ABH, reported feeling that they were partly to blame for their HIV. Overall, these findings demonstrate significant differences within the population of ALHIV and underscore the importance of developing gender sensitive interventions inclusive including stigma mitigation.

Female ABH in our study bear specific attention as they appear to be particularly vulnerable. They were the most socioeconomically disadvantaged with regard to household resources, two-thirds were not in school and half of those no longer in school had never finished primary level. Although we cannot distinguish whether the adolescent females in our cross-sectional study acquired HIV as a result of their vulnerability or whether their HIV status contributed to their poor socioeconomic status, our findings are consistent with previous studies which have identified poverty and lack of education as both drivers of HIV risk and as consequences of HIV infection for adolescent girls in SSA [61–64]. Female ABH in our cohort also had the least knowledge about HIV transmission and viral load testing, and were more likely to say it was acceptable to miss medication doses. Previous studies have also shown that basic HIV knowledge is lower among adolescent girls and young women compared to men [13,65]. It is also important to note that just over half of female ABH in our study had a history of pregnancy, and among those pregnant at the time of the survey, few reported using contraception prior to the pregnancy despite half not intending to get pregnant. Low contraception use and high rates of unintended pregnancies have been reported previously in adolescent populations in SSA, including ALHIV [66,67]. These findings highlight the need to accelerate efforts to improving access to HIV prevention and sexual and reproductive health services for young women in Mozambique.

Our study did not include a comparison group of adolescents who do not have HIV so that we cannot ascertain whether the challenges faced by our cohort differ from those of other young people in Mozambique. The 2015 Mozambique Global School-Based Health Survey of a national representative sample of 16–17 year-old adolescents found similarly low use of alcohol (16%) and other drugs (2%), and a high proportion (64%) of young people reporting sexual activity [68]. Food insecurity and poverty are problems faced not only by ALHIV but widely across the population in Mozambique [69]. Lack of HIV knowledge is also common among ALHIV, particularly young women, and use of contraception is also limited among all adolescents. UNFPA estimates that only 19% of women and girls 15–19 have comprehensive knowledge about HIV [70]. Given the challenges faced by all ALHIV in Mozambique, it is somewhat encouraging that they appear to be similar to their peers in many ways. These findings suggest that interventions targeting all adolescents, in addition to those focused on ALHIV, are needed to improve the health and well-being of young people.

There are few data from SSA with which to compare our findings regarding differences by of HIV transmission mode because only a small number of studies have characterized ALHIV in this way [29–31]. These studies also used different definitions of mode of transmission, mostly ART initiation <10 years as the main variable to distinguish AVH from ABH, whereas we used a cut-off of ART initiation at 15 years in our study. Our decision to use an older age cut-off was based on previous examinations of the ALHIV population showing advanced HIV disease among ALHIV 10–14 years of age suggestive of vertical rather than behavioral acquisition [4,71,72], as well as an investigation of population-level HIV prevalence data from Southern Africa which identified most ALHIV 10–14 years as AVH [7]. No studies, including our own, have included data on transmission mode collected directly from ALHIV or caregivers and instead rely on medical charts which are often missing the relevant data [56,73]. Future research involving ALHIV should include asking participants directly about mode of transmission, and additionally, it would be beneficial for the research community to agree upon a standard definition for using ART and demographic data as a proxy for mode of transmission.

Our analysis is unique in its description of characteristics of ALHIV across multiple domains by mode of transmission and sex. It is also novel for its setting, northern Mozambique, which has a large population of ALHIV about whom there are few data. We recruited participants from routine care settings, including antenatal clinics, to ensure inclusion of female ALHIV in order to more accurately describe the ALHIV population in this setting which is disproportionately female [8]. However, the study was based on a convenience sample of ALHIV actively engaged in care which limits our findings to adolescents in HIV services, the majority of whom were female. A limitation of the study is that we can only describe ALHIV enrolled in HIV services who represent only a portion of the population. In addition, loss to follow-up from care is high among adolescents [3,74] and our study was not designed to examine ALHIV who had disengaged from care nor to measure retention or treatment outcomes. While our aim was to describe ALHIV based on mode of transmission, participants were not asked about this directly and information in medical charts was not available. It is therefore possible that some ALHIV in this analysis were misclassified according to mode of transmission. In addition, because there were so few ABH males in care at the participating health facilities, our ability to identify differences by mode of transmission among males was limited.

In summary, our study provides new data on ALHIV in Mozambique, a country with one of the highest burdens of adolescent HIV in the world. We found that among Mozambican ALHIV engaged in HIV services, there was high level of deprivation with regard to resources, knowledge about HIV, adherence and reproductive health, and a high degree of self-perceived stigma. While the young men and women in the study faced many shared challenges, we also uncovered unique vulnerability by sex and mode of transmission which was particularly pronounced among young women with behaviorally acquired HIV. We believe that these data can contribute to more effective interventions for ALHIV through tailoring service interventions to meet the unique vulnerabilities of this population.

## Supporting information

S1 Fig Social-ecological model (SEM) model taking into account individual-level characteristics and health behaviors, higher-level structural and contextual risk factors that may influence retention in care, ART adherence, and HIV viral suppression for adolescents living with HIV. (TIF)

S2 Fig Estimated mode of transmission among ALHIV 15–19 years of age enrolled in care at three health facilities in Nampula, Mozambique by estimated mode of transmission, 2019 (N = 208). (TIF)

S1 Table Demographic characteristics of adolescents living with HIV 15–19 years of age enrolled in care at three health facilities in Nampula, Mozambique by estimated mode of transmission, 2019 (N = 208). (DOCX)

S2 Table Self-reported health status, adherence and health autonomy among adolescents living with HIV 15–19 years of age care at three health facilities in Nampula, Mozambique by estimated mode of transmission, 2019 (N = 208). (DOCX)

S3 Table Relationship status, HIV stigma/disclosure and community engagement among ALHIV 15–19 years of age enrolled in HIV care in Nampula, Mozambique by estimated mode of transmission, 2019 (N = 208). (DOCX)

S4 Table Social Impact Scale responses from ALHIV 15–19 years of age enrolled in HIV care in Nampula, Mozambique by estimated mode of transmission, 2019 (N = 208). (DOCX)

S5 Table Sexual behaviors and condom use among ALHIV 15–19 years of age who have had vaginal intercourse in Nampula, Mozambique by estimated mode of transmission, 2019 (N = 140). (DOCX)

S1 File (XLSX)
