# Supplementary figures and images for: Characteristics of adolescents aged 15-19 years living with vertically and horizontally acquired HIV in Nampula, Mozambique
Source: PLoS One. 2021 Apr 26;16(4):e0250218. doi: 10.1371/journal.pone.0250218 (PMC8075210; doi:10.1371/journal.pone.0250218)

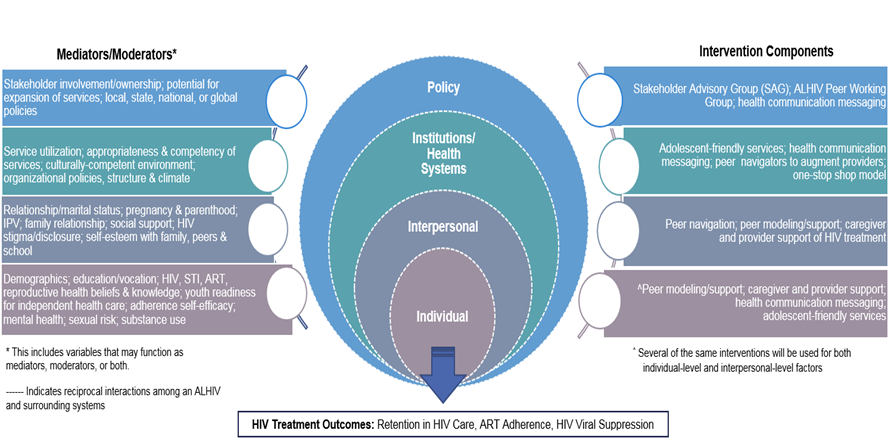

Supplement: S1 Fig — (TIF) [file pone.0250218.s001.tif]

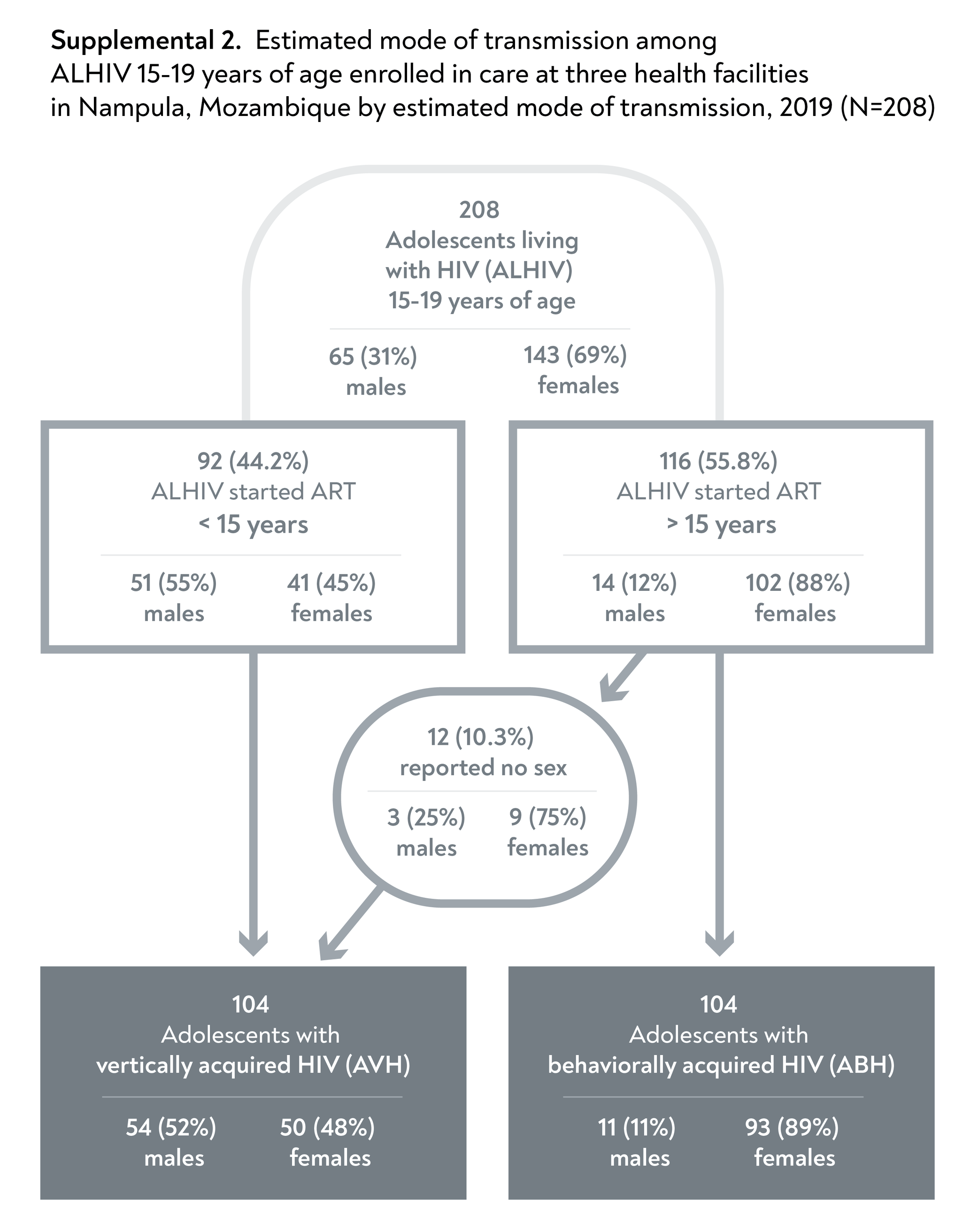

Supplement: S2 Fig — (TIF) [file pone.0250218.s002.tif]
