# Supplementary material for: Characteristics of adolescents aged 15-19 years living with vertically and horizontally acquired HIV in Nampula, Mozambique
Source: PLoS One. 2021 Apr 26;16(4):e0250218. doi: 10.1371/journal.pone.0250218 (PMC8075210; doi:10.1371/journal.pone.0250218)
Supplement: S1 Table — (DOCX) [file pone.0250218.s003.docx]

**Supplemental Table 1.** Demographic characteristics of adolescents living with HIV 15-19 years of age enrolled in care at three health facilities in Nampula, Mozambique by estimated mode of transmission, 2019 (N=208)

|  | **Males** | | | | | **Females** | | | | |
| --- | --- | --- | --- | --- | --- | --- | --- | --- | --- | --- |
|  | **AVH** | | **ABH** | |  | **AVH** | | **ABH** | |  |
|  | **N** | ***%*** | **N** | ***%*** | **p-value** | **N** | ***%*** | **N** | ***%*** | **p-value** |
|  | 54 | *83* | 11 | *17* |  | 50 | *35* | 93 | *65* |  |
| **Age**, median *(IQR)* | 16 *(15-18)* | | 18 *(17-19)* | | <0.01 | 16 *(15-18)* | | 18 *(18-19)* | | <0.001 |
| **Age at ART initiation**, median *(IQR)* | 12 *(8-14)* | | *17 (16-18)* | | <0.001 | 12 *(9-16)* | | 18 *(17-19)* | | <0.001 |
| Don't know | 5 | *9* | 0.0 | *0* | 0.58 | 15 | *30* | 3 | *3* | <0.001 |
| **Household characteristics & resources** |  |  |  |  |  |  |  |  |  |  |
| Muslim | 22 | *41* | 6 | *55* | 0.80 | 14 | *28* | 35 | *38* | 0.38 |
| Catholic | 17 | *32* | 3 | *27* |  | 21 | *42* | 30 | *32* |  |
| Protestant/Evangelic | 14 | *26* | 2 | *18* |  | 14 | *28* | 22 | *24* |  |
| Other / no religion | 1 | *2* | 0 | *0* |  | 1 | *2* | 6 | *7* |  |
| **Primary caregiver** |  |  |  |  |  |  |  |  |  |  |
| Mother | 24 | *44* | 4 | *36* | 0.24 | 22 | *44* | 17 | *18* | <0.001 |
| Father | 8 | *15* | 2 | *18* |  | 7 | *14* | 9 | *10* |  |
| Sister / brother | 7 | *13* | 2 | *18* |  | 7 | *14* | 3 | *3* |  |
| Aunt / uncle | 7 | *13* | 1 | *9* |  | 6 | *12* | 5 | *5* |  |
| Grandmother / grandfather | 7 | *13* | 0 | *0* |  | 4 | *8* | 2 | *2* |  |
| Partner (husband, boyfriend) | 0 | *0* | 0 | *0* |  | 3 | *6* | 28 | *30* |  |
| Self | 0 | *0* | 1 | *9* |  | 1 | *2* | 28 | *30* |  |
| Other | 1 | *2* | 1 | *9* |  | 0 | *0* | 1 | *1* |  |
| **Primary provider of financial support** |  |  |  |  |  |  |  |  |  |  |
| Mother | 13 | *24* | 1 | *9* | 0.14 | 17 | *34* | 15 | *16* | <0.001 |
| Father | 19 | *35* | 2 | *18* |  | 13 | *26* | 12 | *13* |  |
| Other family | 18 | *33* | 6 | *55* |  | 16 | *32* | 3 | *3* |  |
| Partner | 0 | *0* | 0 | *0* |  | 4 | *8* | 55 | *59* |  |
| Self | 0 | *0* | 1 | *9* |  | 0 | *0* | 4 | *4* |  |
| Other | 4 | *7* | 1 | *9* |  | 0 | *0* | 4 | *4* |  |
| **Household facilities** |  |  |  |  |  |  |  |  |  |  |
| Inside toilet | 37 | *70* | 8 | *73* | 1.00 | 14 | *28* | 10 | *11* | <0.01 |
| Running water | 28 | *53* | 2 | *18* | 0.05 | 13 | *26* | 5 | *5* | <0.01 |
| Electricity | 47 | *89* | 8 | *73* | 0.18 | 44 | *88* | 59 | *63* | <0.01 |
| **Adolescent or caregiver can afford** |  |  |  |  |  |  |  |  |  |  |
| Visit to the doctor when you are ill | 52 | *98* | 9 | *82* | 0.07 | 49 | *98* | 91 | *98* | 1.00 |
| Three meals a day | 40 | *76* | 7 | *64* | 0.46 | 42 | *84* | 75 | *81* | 0.62 |
| School fees (n=128) | 47 | *100* | 7 | *88* | 0.15 | 42 | *98* | 27 | *90* | 0.30 |
| All of the medicines needed | 48 | *91* | 8 | *73* | 0.13 | 48 | *96* | 87 | *94* | 0.71 |
| **Not enough food >1 day past week** | 23 | *43* | 8 | *73* | 0.10 | 5 | *10* | 13 | *14* | 0.60 |
| **Cell phone ownership** |  |  |  |  |  |  |  |  |  |  |
| Has own cell phone | 26 | *48* | 4 | *36* | 0.23 | 26 | *52* | 39 | *42* | 0.42 |
| Shares a cell phone | 11 | *20* | 5 | *46* |  | 11 | *22* | 29 | *31* |  |
| No cell phone | 17 | *32* | 2 | *18* |  | 13 | *26* | 25 | *27* |  |
| **Has accessed the internet** | 28 | *52* | 7 | *64* | 0.53 | 24 | *48* | 18 | *19* | <0.001 |
| **Frequency of internet** |  |  |  |  |  |  |  |  |  |  |
| Daily | 13 | *46* | 3 | *43* | 1.00 | 10 | *42* | 6 | *33* | 0.85 |
| Weekly | 12 | *43* | 3 | *43* |  | 10 | *42* | 8 | *44* |  |
| Monthly or more | 3 | *11* | 1 | *14* |  | 4 | *17* | 4 | *22* |  |
| **Substance use (ever)** |  |  |  |  |  |  |  |  |  |  |
| Tobacco | 3 | *6* | 2 | *18* | 0.20 | 0 | *0* | 0 | *0* | - |
| Alcohol | 9 | *17* | 5 | *46* | 0.05 | 4 | *8* | 8 | *9* | 1.00 |
| Marijuana | 2 | *4* | 1 | *9* | 0.43 | 0 | *0* | 0 | *0* | - |
| **Education & employment** |  |  |  |  |  |  |  |  |  |  |
| **Current school enrollment** |  |  |  |  |  |  |  |  |  |  |
| Primary (first and second) | 11 | *20* | 0 | *0* | 0.31 | 6 | *12* | 2 | *2* | <0.001 |
| Secondary (first and second) | 35 | *65* | 8 | *73* |  | 34 | *68* | 29 | *31* |  |
| Technical or vocational school | 1 | *2* | 0 | *0* |  | 1 | *2* | 0 | *0* |  |
| University, college or other tertiary | 1 | *2* | 0 | *0* |  | 1 | *2* | 0 | *0* |  |
| Not in school | 6 | *11* | 3 | *27* |  | 8 | *16* | 62 | *67* |  |
| **Highest grade completed** (n=79) |  |  |  |  |  |  |  |  |  |  |
| None | 0 | *0* | 0 | *0* | 0.64 | 0 | *0* | 4 | *7* | 0.84 |
| Incomplete primary | 4 | *67* | 1 | *33* |  | 4 | *50* | 26 | *42* |  |
| Primary education | 0 | *0* | 0 | *0* |  | 0 | *0* | 7 | *11* |  |
| Incomplete secondary | 1 | *17* | 2 | *67* |  | 3 | *38* | 21 | *34* |  |
| Secondary education | 1 | *17* | 0 | *0* |  | 1 | *13* | 4 | *7* |  |
| **Reasons for not attending school** |  |  |  |  |  |  |  |  |  |  |
| Finished | 1 | *17* | 0 | *0* | 1.00 | 1 | *13* | 1 | *2* | 0.22 |
| Couldn't pay school fee/uniforms | 3 | *50* | 2 | *67* | 1.00 | 0 | *0* | 15 | *24* | 0.19 |
| Stopped to help at home/get job | 0 | *0* | 0 | *0* | - | 1 | *13* | 8 | *13* | 1.00 |
| Too unwell | 0 | *0* | 1 | *33* | 0.33 | 3 | *38* | 2 | *3* | 0.01 |
| Parent/guardian died | 1 | *17* | 0 | *0* | 1.00 | 0 | *0* | 1 | *2* | 1.00 |
| Got married | 0 | *0* | 0 | *0* | - | 0 | *0* | 17 | *27* | 0.19 |
| Pregnancy/had child | 0 | *0* | 0 | *0* | - | 3 | *38* | 22 | *36* | 1.00 |
| Moved | 1 | *17* | 1 | *33* | 1.00 | 1 | *13* | 5 | *8* | 0.53 |
| Other | 1 | *17* | 0 | *0* | 1.00 | 2 | *25* | 11 | *18* | 0.64 |
| **Employment status** |  |  |  |  |  |  |  |  |  |  |
| Currently employed | 1 | *2* | 0 | *0* | 0.01 | 2 | *4* | 4 | *4* | 0.42 |
| Previously employed | 1 | *2* | 3 | *27* |  | 2 | *4* | 10 | *11* |  |
| Never employed | 52 | *96* | 8 | *73* |  | 46 | *92* | 79 | *85* |  |
| **Self-reported health status** |  |  |  |  |  |  |  |  |  |  |
| **Current health status** |  |  |  |  |  |  |  |  |  |  |
| Excellent/very good | 12 | *22* | 2 | *18* | 0.31 | 10 | *20* | 23 | *25* | 0.06 |
| Good | 22 | *41* | 5 | *46* |  | 24 | *48* | 55 | *59* |  |
| Fair | 18 | *33* | 2 | *18* |  | 10 | *20* | 5 | *5* |  |
| Poor | 2 | *4* | 2 | *18* |  | 6 | *12* | 10 | *11* |  |
| **Past year** |  |  |  |  |  |  |  |  |  |  |
| Too sick to attend work/school >1 day | 26 | *48* | 9 | *82* | 0.05 | 23 | *46* | 17 | *18* | <0.001 |
| >1 night in hospital | 6 | *11* | 2 | *18* | 0.61 | 4 | *8* | 9 | *10* | 1.00 |
| TB diagnosis | 6 | *11* | 1 | *9* | 1.00 | 4 | *8* | 3 | *3* | 0.24 |
| **Ever diagnosed or treated for STI** | 2 | *4* | 1 | 9 | 0.43 | 6 | *12* | 24 | *26* | 0.05 |
| **Past 6 months symptoms reported sometimes or often** | | | | |  |  |  |  |  |  |
| Asthma, lung problems and trouble breathing for > 2 days | 11 | *20* | 4 | 36 | 0.26 | 14 | *28* | 15 | *16* | 0.09 |
| Bad cough | 32 | *59* | 4 | 36 | 0.20 | 33 | *66* | 39 | *42* | 0.01 |
| Night sweats | 15 | *28* | 4 | 36 | 0.72 | 14 | *29* | 39 | *42* | 0.12 |
| Ulcers in mouth or problems swallowing food | 12 | *22* | 1 | 9 | 0.44 | 13 | *26* | 21 | *23* | 0.65 |
| Diarrhea >2 days in a row | 28 | *52* | 4 | 36 | 0.51 | 24 | *48* | 38 | *41* | 0.41 |
| Weight loss/inability to put on weight | 20 | *37* | 5 | 46 | 0.74 | 25 | *51* | 43 | *48* | 0.72 |
| Smelly vaginal or penile discharge | 5 | *9* | 0 | 0 | 0.58 | 8 | *16* | 29 | *32* | 0.05 |
| **Medical chart data** |  |  |  |  |  |  |  |  |  |  |
| **CD4 cell count measure past year** | 10 | *19* | 1 | 9 | 0.67 | 7 | *14* | 9 | *10* | 0.43 |
| **Median CD4 cell count** *(IQR)* | 585 *(363, 694)* | | --^ | | 0.75 | 644 *(508, 782)* | | 742 *(606, 954)* | | 0.32 |
| **Viral load measure past year** | 37 | *69* | 4 | 36 | 0.08 | 35 | *70* | 46 | *50* | 0.02 |
| **Median log_10_ viral load** *(IQR)* | 2.9 *(1.7, 4.3)* | | 3.2 *(3.2, 4.4)* | | 0.08 | 1.7 *(1.7, 4.1)* | | 1.7 *(1.7, 3.5)* | | 0.68 |
| <50 copies/mL | 15 | *41* | 2 | *50* | 1.00 | 18 | *51* | 26 | *57* | 0.65 |
| <1000 copies/mL | 20 | *54* | 2 | *50* | 1.00 | 22 | *63* | 31 | *67* | 0.67 |
| **Regimen** (11 missing regimen data) |  |  |  |  |  |  |  |  |  |  |
| TDF+3TC+EFV | 19 | *37* | 5 | *46* | 0.03 | 31 | *66* | 87 | *99* | <0.001 |
| TDF+3TC+DTG | 16 | *31* | 4 | *36* |  | 9 | *19* | 1 | *1* |  |
| 2 NRTI+PI (ATZ/r, LPV/r) | 11 | *22* | 0 | *0* |  | 3 | *6* | 0 | *0* |  |
| AZT+3TC+NVP | 5 | *10* | 0 | *0* |  | 3 | *6* | 0 | *0* |  |
| Other | 0 | *0* | 2 | *18* |  | 1 | *2* | 0 | *0* |  |

*TDF: tenofovir, 3TC: lamivudine, EFV: efavirenz, DTG: dolutegravir, NRT: non-nucleoside reverse transcriptase inhibitors including AZT: zidovudine or ABC: abacavir, ATZ/r: atazanavir/ritonavir, LPV/r: lopinavir/ritonavir, NVP: nevirapine; ^only one male ABH had CD4 count data
