# Supplementary material for: Characteristics of adolescents aged 15-19 years living with vertically and horizontally acquired HIV in Nampula, Mozambique
Source: PLoS One. 2021 Apr 26;16(4):e0250218. doi: 10.1371/journal.pone.0250218 (PMC8075210; doi:10.1371/journal.pone.0250218)
Supplement: S2 Table — (DOCX) [file pone.0250218.s004.docx]

**Supplemental Table 2.** Self-reported health status, adherence and health autonomy among adolescents living with HIV 15-19 years of age care at three health facilities in Nampula, Mozambique by estimated mode of transmission, 2019 (N=208)

|  | **Males** | | | | | **Females** | | | | |
| --- | --- | --- | --- | --- | --- | --- | --- | --- | --- | --- |
|  | **AVH** | | **ABH** | |  | **AVH** | | **ABH** | |  |
|  | **N** | **%** | **N** | **%** | **p-value** | **N** | **%** | **N** | **%** | **p-value** |
|  | 54 | *83* | 11 | *17* |  | 50 | *35* | 93 | *65* |  |
| **ART responsibility** |  |  |  |  |  |  |  |  |  |  |
| **Frequency of ART pick-up** |  |  |  |  |  |  |  |  |  |  |
| Monthly | 37 | *69* | 5 | *46* | 0.31 | 34 | *68* | 85 | *91* | <0.01 |
| Every 3 months | 16 | *30* | 6 | *55* |  | 15 | *30* | 8 | *9* |  |
| Other | 1 | *2* | 0 | *0* |  | 1 | *2* | 0 | *0* |  |
| **Takes one ARV pill per day** | 31 | *57* | 7 | *64* | 0.75 | 37 | *74* | 80 | *86* | 0.08 |
| **Knows prescribed ARVs names** | 2 | *4* | 4 | *36* | 0.01 | 2 | *4* | 0 | *0* | 0.12 |
| **Person responsible for administering ART** | |  |  |  |  |  |  |  |  |  |
| Self | 50 | *93* | 10 | *91* | 0.62 | 44 | *88* | 87 | *94* | 0.45 |
| Caregiver | 2 | *4* | 1 | *9* |  | 3 | *6* | 4 | *4* |  |
| Both self and caregiver | 2 | *4* | 0 | *0* |  | 3 | *6* | 2 | *2* |  |
| **Self-reported ART adherence** |  |  |  |  |  |  |  |  |  |  |
| **Missed ARVs >1 day in past 30 days** | 34 | *63* | 6 | *55* | 0.74 | 20 | *40* | 36 | *39* | 0.88 |
| **Took ART as instructed past 30 days** |  |  |  |  |  |  |  |  |  |  |
| Always | 36 | *67* | 10 | *91* | 0.44 | 25 | *50* | 42 | *45* | 0.04 |
| Almost always | 10 | *19* | 1 | *0* |  | 24 | *48* | 34 | *37* |  |
| Sometimes/usually | 8 | *15* | 0 | *0* |  | 1 | *2* | 10 | *11* |  |
| Never/rarely | 0 | *0* | 0 | *0* |  | 0 | *0* | 7 | *8* |  |
| **Did a good job taking ART as instructed past 30 days** | | |  |  |  |  |  |  |  |  |
| Very poor/poor | 1 | *2* | 0 | *0* | 0.25 | 0 | *0* | 5 | *5* | 0.29 |
| Fair | 9 | *17* | 4 | *36* |  | 4 | *8* | 12 | *13* |  |
| Good | 25 | *46* | 2 | *18* |  | 20 | *40* | 29 | *31* |  |
| Very good/excellent | 19 | *35* | 5 | *46* |  | 26 | *52* | 47 | *51* |  |
| **Difficulty taking ARVs as instructed** |  |  |  |  |  |  |  |  |  |  |
| Not hard/not very hard | 51 | *94* | 11 | *100* | 1.00 | 44 | *88* | 78 | *84* | 0.27 |
| Somewhat hard | 2 | *4* | 0 | *0* |  | 6 | *12* | 10 | *11* |  |
| Extremely hard/very hard | 1 | 2 | 0 | *0* |  | 0 | *0* | 5 | *5* |  |
| **3-item adherence score,** median *(IQR)* | 87 *(81, 92)* | | 88 *(81, 94)* | |  | 89 *(82, 94)* | | 0.35 | | 0.16 |
| **Reported reasons for any missed ART doses past 30 days** | | | |  |  |  |  |  |  |  |
| None missed | 20 | *37* | 5 | *46* | 0.74 | 30 | *60* | 56 | *60* | 0.98 |
| Forgot | 21 | *39* | 4 | *36* | 1.00 | 16 | *32* | 22 | *24* | 0.28 |
| Different routine | 8 | *15* | 3 | *27* | 0.38 | 3 | *6* | 7 | *8* | 1.00 |
| No food to take with ARVs | 6 | *11* | 0 | *0* | 0.58 | 1 | *2* | 8 | *9* | 0.16 |
| Ran out | 3 | *6* | 2 | *18* | 0.20 | 1 | *2* | 2 | *2* | 1.00 |
| Unwell or vomiting | 4 | *7* | 0 | *0* | 1.00 | 1 | *2* | 1 | *1* | 1.00 |
| Didn’t like taste | 2 | *4* | 0 | *0* | 1.00 | 1 | *2* | 2 | *2* | 1.00 |
| Fed up or tired or taking ARVs | 2 | *4* | 0 | *0* | 1.00 | 0 | *0* | 2 | *2* | 0.54 |
| Did not want others to see ARVs | 1 | *2* | 0 | *0* | 1.00 | 1 | *2* | 1 | *1* | 1.00 |
| **Readiness for independent care** |  |  |  |  |  |  |  |  |  |  |
| **Keeps track of clinic appointments** |  |  |  |  |  |  |  |  |  |  |
| Always on own | 40 | *74* | 10 | *91* | 0.53 | 30 | *60* | 74 | *80* | <0.01 |
| With help from caregiver | 8 | *15* | 0 | *0* |  | 10 | *20* | 16 | *17* |  |
| Caregiver always does | 6 | *11* | 1 | *9* |  | 10 | *20* | 3 | *3* |  |
| **Always attends clinic on own** | 44 | *82* | 11 | *100* | 0.19 | 39 | *78* | 86 | *93* | 0.01 |
| **Explain health issues to provider** |  |  |  |  |  |  |  |  |  |  |
| Always on own | 39 | *72* | 10 | *91* | 0.68 | 38 | *76* | 86 | *83* | <0.01 |
| With help from caregiver | 12 | *22* | 1 | *9* |  | 7 | *14* | 7 | *8* |  |
| Caregiver always does | 2 | *4* | 0 | *0* |  | 5 | *10* | 0 | *0* |  |
| N/A (doesn't do) | 1 | *2* | 0 | *0* |  | 0 | *0* | 0 | *0* |  |
| **Tracks stock of own medications** |  |  |  |  |  |  |  |  |  |  |
| Always on own | 18 | *33* | 7 | *64* | 0.12 | 23 | *46* | 63 | *68* | <0.001 |
| With help from caregiver | 14 | *26* | 0 | *0* |  | 10 | *20* | 10 | *11* |  |
| Caregiver always does | 15 | *28* | 2 | *18* |  | 9 | *18* | 1 | *1* |  |
| N/A (doesn't do) | 7 | *13* | 2 | *18* |  | 8 | *16* | 19 | *20* |  |
| **Know when to take medication** |  |  |  |  |  |  |  |  |  |  |
| Always on own | 50 | *93* | 11 | *100* | 1.00 | 47 | *94* | 91 | *98* | 0.25 |
| With help from caregiver | 2 | *4* | 0 | *0* |  | 3 | *6* | 2 | *2* |  |
| Caregiver always does | 2 | *4* | 0 | *0* |  | 0 | *0* | 0 | *0* |  |
