# Supplementary material for: Characteristics of adolescents aged 15-19 years living with vertically and horizontally acquired HIV in Nampula, Mozambique
Source: PLoS One. 2021 Apr 26;16(4):e0250218. doi: 10.1371/journal.pone.0250218 (PMC8075210; doi:10.1371/journal.pone.0250218)
Supplement: S3 Table — (DOCX) [file pone.0250218.s005.docx]

**Supplemental Table 3.** Relationship status, HIV stigma/disclosure and community engagement among ALHIV 15-19 years of age enrolled in HIV care in Nampula, Mozambique by estimated mode of transmission, 2019 (N=208)

|  | **Males** | | | | | **Females** | | | | |
| --- | --- | --- | --- | --- | --- | --- | --- | --- | --- | --- |
|  | **AVH** | | **ABH** | |  | **AVH** | | **ABH** | |  |
|  | **N** | ***%*** | **N** | ***%*** | **p-value** | **N** | ***%*** | **N** | ***%*** | **p-value** |
|  | 54 | *83* | 11 | *17* |  | 50 | *35* | 93 | *65* |  |
| **Current relationship** |  |  |  |  |  |  |  |  |  |  |
| **Currently in a relationship** | 19 | *35* | 4 | *36* | 1.00 | 18 | *36* | 79 | *85* | <0.001 |
| **Current relationship status** |  |  |  |  | - |  |  |  |  | 0.03 |
| Married and living together | 0 | *0* | 0 | *0* |  | 3 | *17* | 39 | *49* |  |
| Married but not living together | 0 | *0* | 0 | *0* |  | 2 | *11* | 13 | *17* |  |
| Not married but living together | 0 | *0* | 0 | *0* |  | 2 | *11* | 5 | *6* |  |
| Not married and not living together | 19 | *100* | 4 | *100* |  | 10 | *56* | 20 | *25* |  |
| Other | 0 | *0* | 0 | *0* |  | 1 | *6* | 2 | *3* |  |
| **Length of current relationship**, median *(IQR)* months | 5 *(2, 12)* | | 8 *(2,12)* | | 0.77 | 12 *(6,36)* | | 12 *(7, 24)* | | 0.65 |
| **Age of partner,** median *(IQR)* age | 15 *(14,17)* | | 19 *(18,19)* | | 0.02 | 22 *(19,23)* | | 24 *(22,27)* | | <0.01 |
| **Age at marriage** (n=57), median *(IQR)* | - | | - | | - | 16 *(15,16)* | | 17 *(16,18)* | | 0.04 |
| **Partner is parent of any of participant's children** (n=58) | 0 | *0* | 0 | *0* | - | 5 | *83* | 43 | *83* | 1.00 |
| **Other sexual partners in last year** (n=111) | 5 | *50* | 1 | *25* | 0.58 | 3 | *17* | 8 | *10* | 0.42 |
| ***HIV status disclosure*** |  |  |  |  |  |  |  |  |  |  |
| **Remembers age when first learned HIV status** | 52 | *96* | 11 | *100* | 1.00 | 42 | *84* | 92 | *99* | 0.00 |
| **Age learned HIV status**, median *(IQR)* | 14 *(13,15)* | | 17 *(16,18)* | | <0.001 | 14 *(12,15)* | | 18 *(17,18)* | | <0.001 |
| **How learned HIV status** |  |  |  |  | 0.01 |  |  |  |  | <0.001 |
| On own (no one told) | 1 | *2* | 0 | *0* |  | 3 | *6* | 1 | *1* |  |
| Told by doctor/nurse | 10 | *19* | 8 | *73* |  | 12 | *24* | 56 | *60* |  |
| Told by family at home | 13 | *24* | 1 | *9* |  | 9 | *18* | 0 | *0* |  |
| Told by family at clinic | 29 | *54* | 2 | *18* |  | 26 | *52* | 35 | *38* |  |
| Don't remember/other | 1 | *2* | 0 | *0* |  | 0 | *0* | 1 | *1* |  |
| **Other household members with HIV** |  |  |  |  |  |  |  |  |  |  |
| Mother | 11 | *20* | 2 | *18* | 1.00 | 24 | *48* | 13 | *14* | <0.001 |
| Father | 4 | *7* | 0 | *0* | 1.00 | 10 | *20* | 6 | *7* | 0.01 |
| Brother/sister | 7 | *13* | 0 | *0* | 0.59 | 15 | *30* | 12 | *13* | 0.01 |
| Aunt/uncle | 9 | *17* | 2 | *18* | 1.00 | 3 | *6* | 6 | *7* | 1.00 |
| Grandmother/grandfather | 6 | *11* | 0 | *0* | 0.58 | 0 | *0* | 0 | *0* | - |
| Partner (wife/husband, boyfriend/girlfriend) | 1 | *2* | 0 | *0* | 1.00 | 1 | *2* | 13 | *14* | 0.02 |
| Other | 21 | *39* | 1 | *9* | 0.08 | 3 | *6* | 16 | *17* | 0.07 |
| Don't know | 12 | *22* | 6 | *55* | 0.06 | 13 | *26* | 41 | *44* | 0.03 |
| **Primary caregiver knows adolescent's HIV status** |  |  |  |  |  |  |  |  |  |  |
| Yes | 52 | *96* | 10 | *91* | 0.19 | 38 | *76* | 40 | *43* | <0.001 |
| No | 0 | *0* | 1 | *9* |  | 10 | *20* | 34 | *37* |  |
| Don't know | 2 | *4* | 0 | *0* |  | 2 | *4* | 19 | *20* |  |
| **Household members know adolescent's HIV status** |  |  |  |  |  |  |  |  |  |  |
| None | 0 | *0* | 1 | *9* | 0.27 | 4 | *8* | 16 | *17* | 0.06 |
| Few | 33 | *61* | 7 | *64* |  | 25 | *50* | 56 | *60* |  |
| Most all | 19 | *35* | 3 | *27* |  | 20 | *40* | 19 | *20* |  |
| Don't know | 2 | *4* | 0 | *0* |  | 1 | *2* | 2 | *2* |  |
| **Family outside household know adolescent's HIV status** |  |  |  |  |  |  |  |  |  |  |
| None | 22 | *41* | 3 | *27* | 0.60 | 16 | *32* | 44 | *47* | 0.14 |
| Few | 22 | *41* | 7 | *64* |  | 24 | *48* | 40 | *43* |  |
| Most all | 6 | *11* | 1 | *9* |  | 6 | *12* | 7 | *8* |  |
| Don't know | 4 | *7* | 0 | *0* |  | 4 | *8* | 2 | *2* |  |
| **Disclosed HIV status to current partner** among those with partners (n=120) | 1 | *5* | 2 | *50* | 0.07 | 8 | *44* | 59 | *75* | 0.01 |
| **Knows current partner's HIV status** (n=120) | 1 | *5* | 2 | *50* | 0.07 | 8 | *44* | 45 | *57* | 0.34 |
| **Current partner's HIV status** (n=56) |  |  |  |  |  |  |  |  |  |  |
| HIV-positive | 1 | *100* | 1 | *50* | 1.00 | 1 | *13* | 19 | *42* | 0.23 |
| HIV-negative | 0 | *0* | 1 | *50* |  | 7 | *88* | 26 | *58* |  |
| **Friends know adolescent's HIV status** |  |  |  |  |  |  |  |  |  |  |
| None | 45 | *83* | 10 | *91* | 1.00 | 45 | *90* | 84 | *90* | 0.27 |
| Few | 7 | *13* | 1 | *9* |  | 2 | *4* | 6 | *7* |  |
| Most all | 0 | *0* | 0 | *0* |  | 2 | *4* | 0 | *0* |  |
| Don't know | 2 | *4* | 0 | *0* |  | 1 | *2* | 3 | *3* |  |
| **Teachers know adolescent's HIV status** among those in school (n=127) |  |  |  |  |  |  |  |  |  |  |
| None | 45 | *94* | 7 | *88* | 0.47 | 41 | *95* | 28 | *100* | 0.52 |
| Some | 3 | *6* | 1 | *13* |  | 2 | *5* | 0 | *0* |  |
| **Community engagement** |  |  |  |  |  |  |  |  |  |  |
| **Member of a youth organization(s)** |  |  |  |  |  |  |  |  |  |  |
| Gospel choir / church group | 3 | *6* | 3 | *27* | 0.06 | 15 | *31* | 16 | *18* | 0.08 |
| Activist organization/youth association | 13 | *25* | 1 | *9* | 0.43 | 2 | *4* | 2 | *2* | 0.61 |
| Sports team | 8 | *15* | 1 | *9* | 1.00 | 2 | *4* | 0 | *0* | 0.12 |
| Music/singing/arts performance group | 2 | *4* | 0 | *0* | 1.00 | 5 | *10* | 1 | *1* | 0.02 |
| Any above | 22 | *42* | 4 | *36* | 1.00 | 20 | *41* | 19 | *21* | 0.01 |
| **Past year involved/participated in following:** |  |  |  |  |  |  |  |  |  |  |
| Cultural or religious organization | 20 | *38* | 4 | *36* | 1.00 | 27 | *55* | 20 | *22* | <0.001 |
| Sports or Recreation | 28 | *53* | 5 | *46* | 0.75 | 8 | *16* | 3 | *3* | 0.02 |
| Performing Arts | 11 | *21* | 3 | *27* | 0.69 | 15 | *31* | 11 | *12* | 0.01 |
| Academic/Pre-Professional Society | 2 | *4* | 1 | *9* | 0.44 | 2 | *4* | 4 | *4* | 1.00 |
| Government or Political Org | 0 | *0* | 0 | *0* | - | 1 | *2* | 3 | *3* | 1.00 |
| Community Based Organization | 1 | *2* | 1 | *9* | 0.32 | 0 | *0* | 3 | *3* | 0.55 |
| Media (newspaper, radio, TV, etc.) | 2 | *4* | 3 | *27* | 0.03 | 2 | *4* | 1 | *1* | 0.28 |
| Other | 0 | *0* | 0 | *0* | - | 0 | *0* | 1 | *1* | 1.00 |
