# Supplementary material for: Characteristics of adolescents aged 15-19 years living with vertically and horizontally acquired HIV in Nampula, Mozambique
Source: PLoS One. 2021 Apr 26;16(4):e0250218. doi: 10.1371/journal.pone.0250218 (PMC8075210; doi:10.1371/journal.pone.0250218)
Supplement: S4 Table — (DOCX) [file pone.0250218.s006.docx]

**Supplemental Table 4**. Social Impact Scale responses from ALHIV 15-19 years of age enrolled in HIV care in Nampula, Mozambique by estimated mode of transmission, 2019 (N=208)

|  | **Males** | | | | | | **Females** | | | | | | **P-value** | | |
| --- | --- | --- | --- | --- | --- | --- | --- | --- | --- | --- | --- | --- | --- | --- | --- |
|  | **AVH** | | **ABH** | | **All** | | **AVH** | | **ABH** | | **All** | | **males vs. females** | **AVH vs. ABH males** | **AVH vs. ABH females** |
|  | **n** | **%** | **n** | **%** | **n** | **%** | **n** | **%** | **n** | **%** | **n** | **%** |  |  |  |
|  | 54 | 83.1 | 11 | 16.9 | 65 | 31.3 | 50 | 35.0 | 93 | 65.0 | 143 | 68.8 |  |  |  |
| ***My teachers or employers have treated me badly because of my HIV, among those in school or employed*** |  |  |  |  |  |  |  |  |  |  |  |  |  |  |  |
| Strongly agree/agree | 1 | 2.0 | 0 | 0.0 | 1 | 1.8 | 0 | 0.0 | 0 | 0.0 | 0 | 0.0 | 0.029 | 0.657 | 0.056 |
| Strongly disagree/disagree | 7 | 14.3 | 2 | 25.0 | 9 | 15.8 | 5 | 11.1 | 0 | 0.0 | 5 | 5.6 |  |  |  |
| Haven't disclosed | 41 | 83.7 | 6 | 75.0 | 47 | 82.5 | 40 | 88.9 | 44 | 100.0 | 84 | 94.4 |  |  |  |
| ***Some people act as though I can't do things that I should be able to do*** |  |  |  |  |  |  |  |  |  |  |  |  |  |  |  |
| Strongly agree/agree | 18 | 33.3 | 2 | 18.2 | 20 | 30.8 | 8 | 16.0 | 4 | 4.3 | 12 | 8.4 | <0.001 | 0.266 | 0.030 |
| Strongly disagree/disagree | 33 | 61.1 | 7 | 63.6 | 40 | 61.5 | 16 | 32.0 | 25 | 26.9 | 41 | 28.7 |  |  |  |
| Haven't disclosed | 3 | 5.6 | 2 | 18.2 | 5 | 7.7 | 26 | 52.0 | 64 | 68.9 | 90 | 62.9 |  |  |  |
| ***I feel I have been treated with less respect than usual by others*** |  |  |  |  |  |  |  |  |  |  |  |  |  |  |  |
| Strongly agree/agree | 21 | 38.9 | 3 | 27.3 | 24 | 36.9 | 3 | 6.0 | 6 | 6.5 | 9 | 6.3 | <0.001 | 0.359 | 0.584 |
| Strongly disagree/disagree | 32 | 59.3 | 7 | 63.6 | 39 | 60.0 | 22 | 44.0 | 33 | 35.5 | 55 | 38.5 |  |  |  |
| Haven't disclosed | 1 | 1.9 | 1 | 9.1 | 2 | 3.1 | 25 | 50.0 | 54 | 58.1 | 79 | 55.2 |  |  |  |
| ***I feel others are worried that they could "catch" my HIV by shaking my hand or sharing food with me*** |  |  |  |  |  |  |  |  |  |  |  |  |  |  |  |
| Strongly agree/agree | 10 | 18.5 | 0 | 0.0 | 10 | 15.4 | 4 | 8.0 | 3 | 3.2 | 7 | 4.9 | <0.001 | 0.179 | 0.498 |
| Strongly disagree/disagree | 42 | 77.8 | 10 | 90.9 | 52 | 80.0 | 17 | 34.0 | 33 | 35.5 | 50 | 35.0 |  |  |  |
| Haven't disclosed | 2 | 3.7 | 1 | 9.1 | 3 | 4.6 | 29 | 58.0 | 57 | 61.3 | 86 | 60.1 |  |  |  |
| ***Health care workers at the clinic treat me badly because of my HIV*** |  |  |  |  |  |  |  |  |  |  |  |  |  |  |  |
| Strongly agree/agree | 0 | 0.0 | 0 | 0.0 | 0 | 0.0 | 0 | 0.0 | 1 | 1.1 | 1 | 0.7 | <0.001 | - | 0.591 |
| Strongly disagree/disagree | 54 | 100.0 | 11 | 100.0 | 65 | 100.0 | 43 | 86.0 | 72 | 77.4 | 115 | 80.4 |  |  |  |
| Haven't disclosed | 0 | 0.0 | 0 | 0.0 | 0 | 0.0 | 7 | 14.0 | 20 | 21.5 | 27 | 18.9 |  |  |  |
| ***I feel others avoid me because of my HIV*** |  |  |  |  |  |  |  |  |  |  |  |  |  |  |  |
| Strongly agree/agree | 6 | 11.1 | 0 | 0.0 | 6 | 9.2 | 5 | 10.0 | 6 | 6.5 | 11 | 7.7 | <0.001 | 0.021 | 0.737 |
| Strongly disagree/disagree | 48 | 88.9 | 9 | 81.8 | 57 | 87.7 | 18 | 36.0 | 33 | 35.5 | 51 | 35.7 |  |  |  |
| Haven't disclosed | 0 | 0.0 | 2 | 18.2 | 2 | 3.1 | 27 | 54.0 | 54 | 58.1 | 81 | 56.6 |  |  |  |
| ***I feel some family members have rejected me because of my HIV*** |  |  |  |  |  |  |  |  |  |  |  |  |  |  |  |
| Strongly agree/agree | 2 | 3.7 | 0 | 0.0 | 2 | 3.1 | 1 | 2.0 | 7 | 7.5 | 8 | 5.6 | <0.001 | 0.192 | 0.181 |
| Strongly disagree/disagree | 52 | 96.3 | 10 | 90.9 | 62 | 95.4 | 34 | 68.0 | 50 | 53.8 | 84 | 58.7 |  |  |  |
| Haven't disclosed | 0 | 0.0 | 1 | 9.1 | 1 | 1.5 | 15 | 30.0 | 36 | 38.7 | 51 | 35.7 |  |  |  |
| ***I feel some friends have rejected me because of my HIV*** |  |  |  |  |  |  |  |  |  |  |  |  |  |  |  |
| Strongly agree/agree | 3 | 5.6 | 0 | 0.0 | 3 | 4.6 | 1 | 2.0 | 3 | 3.3 | 4 | 2.8 | 0.001 | 1.000 | 1.000 |
| Strongly disagree/disagree | 15 | 27.8 | 3 | 27.3 | 18 | 27.7 | 4 | 8.0 | 8 | 8.7 | 12 | 8.5 |  |  |  |
| Haven't disclosed | 36 | 66.7 | 8 | 72.7 | 44 | 67.7 | 45 | 90.0 | 81 | 88.0 | 126 | 88.7 |  |  |  |
| ***I encounter embarrassing situations as a result of my HIV*** |  |  |  |  |  |  |  |  |  |  |  |  |  |  |  |
| Strongly agree/agree | 14 | 25.9 | 0 | 0.0 | 14 | 21.5 | 11 | 22.0 | 25 | 27.5 | 36 | 25.5 | <0.001 | 0.200 | 0.592 |
| Strongly disagree/disagree | 39 | 72.2 | 11 | 100.0 | 50 | 76.9 | 25 | 50.0 | 37 | 40.7 | 62 | 44.0 |  |  |  |
| Haven't disclosed | 1 | 1.9 | 0 | 0.0 | 1 | 1.5 | 14 | 28.0 | 29 | 31.9 | 43 | 30.5 |  |  |  |
| ***Due to my HIV others seem to feel awkward and tense when they are around me*** |  |  |  |  |  |  |  |  |  |  |  |  |  |  |  |
| Strongly agree/agree | 7 | 13.0 | 0 | 0.0 | 7 | 10.8 | 0 | 0.0 | 5 | 5.4 | 5 | 3.5 | <0.001 | 0.544 | 0.199 |
| Strongly disagree/disagree | 45 | 83.3 | 11 | 100.0 | 56 | 86.2 | 20 | 40.0 | 29 | 31.2 | 49 | 34.3 |  |  |  |
| Haven't disclosed | 2 | 3.7 | 0 | 0.0 | 2 | 3.1 | 30 | 60.0 | 59 | 63.4 | 89 | 62.2 |  |  |  |
| ***I feel others think I am to blame for my HIV*** |  |  |  |  |  |  |  |  |  |  |  |  |  |  |  |
| Strongly agree/agree | 3 | 5.6 | 1 | 9.1 | 4 | 6.2 | 4 | 8.0 | 8 | 8.6 | 12 | 8.4 | <0.001 | 0.801 | 0.929 |
| Strongly disagree/disagree | 47 | 87.0 | 9 | 81.8 | 56 | 86.2 | 25 | 50.0 | 42 | 45.2 | 67 | 46.9 |  |  |  |
| Haven't disclosed | 4 | 7.4 | 1 | 9.1 | 5 | 7.7 | 21 | 42.0 | 43 | 46.2 | 64 | 44.8 |  |  |  |
| ***I do not feel I can be open with others about my HIV*** |  |  |  |  |  |  |  |  |  |  |  |  |  |  |  |
| Strongly agree/agree | 13 | 24.1 | 3 | 27.3 | 16 | 24.6 | 19 | 38.8 | 39 | 41.9 | 58 | 40.9 | <0.001 | 1.000 | 0.786 |
| Strongly disagree/disagree | 41 | 75.9 | 8 | 72.7 | 49 | 75.4 | 13 | 26.5 | 27 | 29.0 | 40 | 28.2 |  |  |  |
| Haven't disclosed | 0 | 0.0 | 0 | 0.0 | 0 | 0.0 | 17 | 34.7 | 27 | 29.0 | 44 | 31.0 |  |  |  |
| ***I fear someone telling others about my HIV without my permission*** |  |  |  |  |  |  |  |  |  |  |  |  |  |  |  |
| Strongly agree/agree | 23 | 42.6 | 4 | 36.4 | 27 | 41.5 | 30 | 60.0 | 51 | 55.4 | 81 | 57.0 | <0.001 | 0.614 | 0.148 |
| Strongly disagree/disagree | 29 | 53.7 | 6 | 54.6 | 35 | 53.9 | 12 | 24.0 | 14 | 15.2 | 26 | 18.3 |  |  |  |
| Haven't disclosed | 2 | 3.7 | 1 | 9.1 | 3 | 4.6 | 8 | 16.0 | 27 | 29.4 | 35 | 24.7 |  |  |  |
| ***I feel I need to keep my HIV a secret*** |  |  |  |  |  |  |  |  |  |  |  |  |  |  |  |
| Strongly agree/agree | 47 | 87.0 | 9 | 81.8 | 56 | 86.2 | 36 | 72.0 | 66 | 71.0 | 102 | 71.3 | <0.001 | 0.642 | 0.469 |
| Strongly disagree/disagree | 7 | 13.0 | 2 | 18.2 | 9 | 13.9 | 8 | 16.0 | 10 | 10.8 | 18 | 12.6 |  |  |  |
| Haven't disclosed | 0 | 0.0 | 0 | 0.0 | 0 | 0.0 | 6 | 12.0 | 17 | 18.3 | 23 | 16.1 |  |  |  |
| ***I feel I am at least partially to blame for my HIV*** |  |  |  |  |  |  |  |  |  |  |  |  |  |  |  |
| Strongly agree/agree | 35 | 64.8 | 9 | 81.8 | 44 | 67.7 | 8 | 16.3 | 15 | 16.3 | 23 | 16.3 | <0.001 | 0.480 | 0.547 |
| Strongly disagree/disagree | 19 | 35.2 | 2 | 18.2 | 21 | 32.3 | 36 | 73.5 | 61 | 66.3 | 97 | 68.8 |  |  |  |
| Haven't disclosed | 0 | 0.0 | 0 | 0.0 | 0 | 0.0 | 5 | 10.2 | 16 | 17.4 | 21 | 14.9 |  |  |  |
