# Supplementary material for: Characteristics of adolescents aged 15-19 years living with vertically and horizontally acquired HIV in Nampula, Mozambique
Source: PLoS One. 2021 Apr 26;16(4):e0250218. doi: 10.1371/journal.pone.0250218 (PMC8075210; doi:10.1371/journal.pone.0250218)
Supplement: S5 Table — (DOCX) [file pone.0250218.s007.docx]

**Supplemental Table 5.** Sexual behaviors and condom use among ALHIV 15-19 years of age who have had vaginal intercourse in Nampula, Mozambique by estimated mode of transmission, 2019 (N=140)

|  | **Males** | | | | | **Females** | | | | |
| --- | --- | --- | --- | --- | --- | --- | --- | --- | --- | --- |
|  | **AVH** | | **ABH** | |  | **AVH** | | **ABH** | |  |
|  | **N** | ***%*** | **N** | ***%*** | **p-value** | **N** | ***%*** | **N** | ***%*** | **p-value** |
|  | 22 | *67* | 11 | *33* |  | 14 | *13* | 93 | *87* |  |
| **Portion of all ALHIV in survey who had sex** | 22 | *41* | 11 | *100* | 0.01 | 14 | *28* | 93 | *100* | <0.0001 |
| ***Sexual behavior history*** |  |  |  |  |  |  |  |  |  |  |
| **Age at first sex,** median *(IQR)*age | 14 *(13,16)* | | 16 *(15,17)* | | <0.01 | 17 *(15,18)* | | 16 *(15,17)* | | 0.33 |
| **Age of first sexual partner,** median *(IQR)* | 14 *(13,15)* | | 16 *(15,18)* | | 0.00 | 19 *(17,20)* | | 19 *(18,22)* | | 0.39 |
| **Forced first sex (all)** | 6 | *27* | 2 | *18* | 0.69 | 2 | *14* | 20 | *22* | 0.73 |
| **Forced first sex** |  |  |  |  | 1.00 |  |  |  |  | 0.12 |
| Physically forced | 2 | *33* | 0 | *0* |  | 2 | *100* | 6 | *30* |  |
| Pressured | 4 | *67* | 2 | *100* |  | 0 | *0* | 14 | *70* |  |
| **Reason had first sex** |  |  |  |  |  |  |  |  |  |  |
| Wanted to try it | 12 | *55* | 6 | *55* | 0.25 | 6 | *43* | 51 | *55* | 0.61 |
| Partner wanted to have sex | 5 | *23* | 3 | *27* |  | 2 | *14* | 13 | *14* |  |
| To show love/to feel loved | 2 | *9* | 0 | *0* |  | 3 | *21* | 18 | *19* |  |
| Pressure from friends | 3 | *14* | 0 | *0* |  | 1 | *7* | 2 | *2* |  |
| For money or gifts | 0 | *0* | 2 | *18* |  | 0 | *0* | 0 | *0* |  |
| Wanted to have a baby | 0 | *0* | 0 | *0* |  | 0 | *0* | 2 | *2* |  |
| Other/don’t know | 0 | *0* | 0 | *0* |  | 2 | *14* | 7 | *8* |  |
| **Total number of sex partners**, median *(IQR)* | 3 *(2,5)* | | 2 *(1,4)* | | 0.33 | 2 *(1,3)* | | 2 *(1,3)* | | 0.61 |
| **Number sex partners last 12 months**, median *(IQR)* | 1 *(0,2)* | | 1 *(0,2)* | | 0.67 | 1 *(1,1)* | | 1 *(1,1)* | | 0.94 |
| **Sex in exchange for money or gifts** | 2 | *9* | 2 | *18* | 0.59 | 3 | *21* | 13 | *14* | 0.44 |
| **Sources of information about sex and reproductive health** |  |  |  |  |  |  |  |  |  |  |
| Friends | 11 | *50* | 8 | *73* | 0.28 | 5 | *36* | 43 | *46* | 0.57 |
| Someone at the clinic | 10 | *46* | 4 | *36* | 0.72 | 6 | *43* | 47 | *51* | 0.59 |
| School | 12 | *55* | 3 | *27* | 0.27 | 4 | *29* | 18 | *19* | 0.48 |
| Internet | 9 | *41* | 5 | *46* | 1.00 | 3 | *21* | 7 | *8* | 0.12 |
| Caregiver | 3 | *14* | 0 | *0* | 0.53 | 1 | *7* | 8 | *9* | 1.00 |
| The media | 2 | *9* | 0 | *0* | 0.54 | 2 | *14* | 7 | *8* | 0.33 |
| Older siblings | 8 | *36* | 0 | *0* | 0.03 | 1 | *7* | 2 | *2* | 0.35 |
| Other | 0 | *0* | 1 | *9* | 0.33 | 3 | *21* | 5 | *5* | 0.07 |
| ***Condom use*** |  |  |  |  |  |  |  |  |  |  |
| **Condom used last sex** | 14 | *64* | 6 | *55* | 0.71 | 8 | *62* | 19 | *20* | <0.01 |
| **Reasons for not using condom last sex act** (n=93) |  |  |  |  |  |  |  |  |  |  |
| Partner refused | 0 | *0* | 1 | *20* | 0.39 | 4 | *67* | 55 | *74* | 0.65 |
| Did not have a condom | 5 | *63* | 2 | *40* | 0.59 | 2 | *33* | 11 | *15* | 0.25 |
| Felt safe without a condom | 0 | *0* | 0 | *0* | - | 1 | *17* | 6 | *8* | 0.43 |
| Wanted to become pregnant or get my partner pregnant | 0 | *0* | 0 | *0* | - | 0 | *0* | 6 | *8* | 1.00 |
| Other | 3 | *38* | 2 | *40* | 1.00 | 1 | *17* | 5 | *7* | 0.38 |
| **Condom use** |  |  |  |  |  |  |  |  |  |  |
| Always | 12 | *55* | 3 | *27* | 0.20 | 6 | *43* | 5 | *5* | <0.001 |
| Sometimes | 4 | *18* | 4 | *36* |  | 5 | *36* | 34 | *37* |  |
| Never | 6 | *27* | 3 | *27* |  | 2 | *14* | 53 | *57* |  |
| Don’t remember | 0 | *0* | 1 | *9* |  | 1 | *7* | 1 | *1* |  |
| **Reasons for condom use over past year** (n=140) |  |  |  |  |  |  |  |  |  |  |
| Prevent pregnancy | 8 | *36* | 3 | *27* | 0.71 | 9 | *64* | 15 | *16* | <0.001 |
| Prevent HIV transmission | 9 | *41* | 1 | *9* | 0.11 | 6 | *43* | 13 | *14* | 0.01 |
| Prevent getting infected with STIs | 4 | *18* | 4 | *36* | 0.39 | 5 | *36* | 7 | *8* | 0.01 |
| Prevent reinfection with HIV | 2 | *9* | 2 | *18* | 0.59 | 3 | *21* | 6 | *7* | 0.09 |
| Other/don't know | 3 | *14* | 1 | *9* | 1.00 | 3 | *21* | 26 | *28* | 0.75 |
| No condom use last year | 7 | *32* | 4 | *36* | 1.00 | 1 | *7* | 40 | *43* | 0.02 |
